# Synaptic Involvement of the Human Amygdala in Parkinson’s Disease

**DOI:** 10.1016/j.mcpro.2023.100673

**Published:** 2023-10-29

**Authors:** Sandra Villar-Conde, Veronica Astillero-Lopez, Melania Gonzalez-Rodriguez, Daniel Saiz-Sanchez, Alino Martinez-Marcos, Isabel Ubeda-Banon, Alicia Flores-Cuadrado

**Affiliations:** 1 Grupo de Neuroplasticidad y Neurodegeneración, CRIB, Facultad de Medicina de Ciudad Real, Universidad de Castilla-La Mancha (UCLM), Spain; 2 Grupo de Neuroplasticidad y Neurodegeneración, Instituto de Investigación Sanitaria de Castilla-La Mancha (IDISCAM), Spain

**Keywords:** limbic system, movement disorders, nonmotor symptoms, pathophysiology, proteinopathy, stromal cell-derived receptor 1 (SDR-1), leukocyte receptor cluster member 7 (LENG7), alpha-1-acid glycoprotein 2 (AGP2), gene associated with retinoic and interferon-induced mortality 19 protein (GRIM19)

## Abstract

α-Synuclein, a protein mostly present in presynaptic terminals, accumulates neuropathologically in Parkinson’s disease in a 6-stage sequence and propagates in the nervous system in a prion-like manner through neurons and glia. In stage 3, the substantia nigra are affected, provoking motor symptoms and the amygdaloid complex, leading to different nonmotor symptoms; from here, synucleinopathy spreads to the temporal cortex and beyond. The expected increase in Parkinson's disease incidence accelerates the need for detection biomarkers; however, the heterogeneity of this disease, including pathological aggregates and pathophysiological pathways, poses a challenge in the search for new therapeutic targets and biomarkers. Proteomic analyses are lacking, and the literature regarding synucleinopathy, neural and glial involvement, and volume of the human amygdaloid complex is controversial. Therefore, the present study combines both proteomic and stereological probes. Data-independent acquisition-parallel accumulation of serial fragmentation proteomic analysis revealed a remarkable proteomic impact, especially at the synaptic level in the human amygdaloid complex in Parkinson’s disease. Among the 199 differentially expressed proteins, guanine nucleotide-binding protein G(i) subunit alpha-1 (GNAI1), elongation factor 1-alpha 1 (EEF1A1), myelin proteolipid protein (PLP1), neuroplastin (NPTN), 14-3-3 protein eta (YWHAH), gene associated with retinoic and interferon-induced mortality 19 protein (GRIM19), and orosomucoid-2 (ORM2) stand out as potential biomarkers in Parkinson’s disease. Stereological analysis, however, did not reveal alterations regarding synucleinopathy, neural or glial populations, or volume changes. To our knowledge, this is the first proteomic study of the human amygdaloid complex in Parkinson’s disease, and it identified possible biomarkers of the disease. Lewy pathology could not be sufficient to cause neurodegeneration or alteration of microglial and astroglial populations in the human amygdaloid complex in Parkinson’s disease. Nevertheless, damage at the proteomic level is manifest, showing up significant synaptic involvement.

## Highlights

• First proteomic and stereological study in the human in Parkinson's disease.
• Lewy pathology might not be sufficient to cause neurodegeneration.
• Synaptic involvement was demonstrated by proteomic data.

## Graphical Abstract

**Figure:**
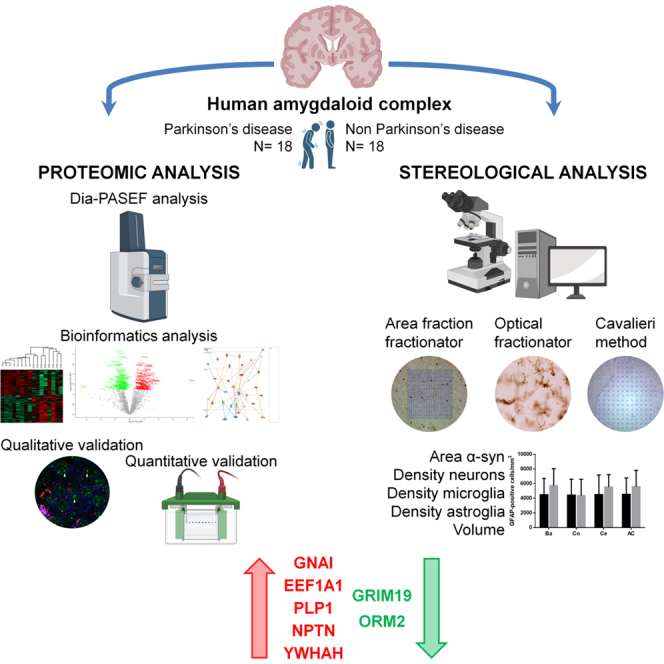

## In Brief

Regarding human amygdaloid complex involvement in Parkinson’s disease, proteomic analyses are lacking, and the literature about synucleinopathy, neural, and glial contribution and volume reduction is controversial. Proteomic analysis revealed a remarkable synaptic involvement, including proteins such as GNAI1, EEF1A1, PLP1, NPTN, YWHAH, GRIM19, and ORM2. No alterations in synucleinopathy, neural, or glial populations or volume changes were observed by stereology. Lewy pathology could not be sufficient to cause neurodegeneration or neuroinflammation but induces a synaptic damage.

Parkinson's disease (PD) is the second most common neurodegenerative disease and is characterized by nigrostriatal denervation caused by the death of dopaminergic neurons in the substantia nigra, provoking motor symptoms that lead to clinical diagnosis (bradykinesia, rigidity, resting tremor, and gait alterations). In parallel, and often decades before, pathological involvement of many other structures leads to prodromal, nonmotor symptoms (hyposmia, sleep disorders, neuropsychiatric features, autonomic dysfunction, mild cognitive impairment and pain, and cognitive disturbances) (1, 2, 3). This disease is included in synucleinopathies due to the involvement of misfolding and aggregation of α-synuclein (α-syn). This proteinopathy is cumulative and predictable allowing staging and pathological diagnosis (4). Interestingly, stage 3 is characterized not only by the involvement of the substantia nigra causing motor symptoms but also by the involvement of the amygdaloid complex (AC), which provokes hyposmia (5), dysautonomia, apathy, anxiety, and depression (6, 7) and facilitates synucleinopathy progression to the temporal cortex and beyond causing dementia (8, 9).

α-Synucleinopathy is characterized by aggregates constituting Lewy bodies (LBs) and neurites (LNs) (10). This protein (α-syn), encoded by the *SNCA* gene, is highly expressed throughout the brain and is particularly enriched in the presynaptic terminals of neurons. In physiological conditions, α-syn is a synaptic vesicle-bound multimer, whereas in diseased conditions, it is able to modify its conformation forming deposits (11). Furthermore, these misfolded proteins can induce naïve proteins to misfold (seeding) and propagate (spreading) (12) through the nervous system in a prion-like manner through synaptic connections (13). Although spreading mainly occurs through neurons, it has been proposed that α-syn toxicity includes neuroinflammation, where α-syn could be a primary trigger for this phenomenon (13). The earliest studies revealed the presence of astrocytes loaded with α-syn aggregates in the AC (14, 15) and microglia activation in PD (16). Whether astroglia and microglia facilitate the clearance of α-syn or, conversely, facilitate its spread (17, 18, 19, 20) remains unclear. However, the implications for the glial population are not known.

The expected increase in the incidence of PD estimated for the coming years (21) raises the urgency to find treatments that stop or delay the disease. However, the heterogeneity of PD, including clinical phenotypes, genetic predispositions, pathological aggregates, and pathophysiological pathways, poses a challenge in the search for new therapeutic targets and biomarkers (22). Therefore, it would be interesting to carry out proteomic analysis on potential proteins that interact with α-syn or synaptic proteins to try to identify pathophysiological pathways and potential biomarkers. Available proteomic studies have focused on the substantia nigra, but essential hubs, such as the AC, have not been investigated (23).

On the other hand, examination of neurodegeneration using specific neural markers has not been carried out, and the glial population has not been stereologically quantified. The AC, as mentioned above, is preferentially involved in pathology. It is a heterogeneous and intricate structure with multiple cortical and subcortical connections (24). Historically, a differential distribution of LBs and LNs has been qualitatively described in this nuclear complex, with the accessory cortical and central nucleus being the most involved in pathology (25, 26), although quantitative data have not confirmed these observations (26). This apparent differential affectation could be explained according to the prion-like hypothesis (13). The cortical nucleus has projections with the olfactory regions, the central nucleus has connections with the autonomic nuclei of the brainstem, and the basal and lateral nuclei have connections with the limbic system and neocortex (27). The greater presence of LBs in the cortical nucleus was shown to be related to its neurodegeneration and loss of volume in a unique stereological study published on this topic (28). Hence, the possible volume reduction of the human AC with PD could be a biomarker. However, the results of volume quantification in patient (29, 30, 31, 32) studies are highly contradictory. Postmortem estimation using the stereological Cavalieri method could provide a more precise approximation since it makes it possible to delimit the different nuclei of the AC.

For all these reasons, the present study consists of a complementary proteomic and stereological analysis of the human AC. Proteomic analysis is used to identify the main protein alterations of the human AC in PD related to α-syn, neuroinflammation, and synapses that can act as biochemical biomarkers of the disease. Stereological analysis is used to analyze the fraction of the area occupied by α-syn in the main nuclei of the AC in PD and its impact on specifically identified neuronal, microglial, and astroglial populations, as well as possible volumetric changes of its anatomical structure that can be used as imaging biomarkers. Therefore, this report constitutes the first proteomic and stereological study analyzing neuronal and glial populations using specific markers in the human AC in PD.

## Experimental Procedures

### Experimental Design and Statistical Rationale

All experiments were performed in accordance with the Declaration of Helsinki principles and the Clinical Research Ethics Committee of the University Hospital of Ciudad Real (PID2019-108659RB-I00).

Thirty-six *postmortem amygdala* from PD and non-PD (NPD) patients were used: 16 frozen unfixed cases (n = 8 PD, n = 8 NPD) for proteomic analysis (Table 1) and 20 fixed cases (n = 10 PD, n = 10 NPD) for stereological analysis (Table 2). First, data-independent acquisition-parallel accumulation serial fragmentation (dia-PASEF) was carried out by the *Instituto Maimonides de Investigación Biomédica de Córdoba* IMIBIC Proteomic Facility. Second, for the bioinformatic study, different tools were used such as Perseus, Biological General Repository for Interaction Datasets (BioGRID), Synaptic Gene Ontologies (SynGO), and Ingenuity (IPA), to identify proteins, interactors with α-syn, differentially expressed proteins (DEPs) in synapses and their role, and biofunctional pathways and protein‒protein interactions, respectively. After that, some DEPs were validated by immunofluorescence (fixed samples) and Western blot (frozen samples).

**Table 1:** Demographic, clinicopathological features, and assay of the cases used for proteomic analysis

Diagnosis	Case	Assay	Braak syn stage	Braak tau stage	Age (years)	Sex	Duration of PD (years)	PMD (hh:mm)	Autopsy brain weight (g)	Autopsy date	Reception date	Processing date	Sample storage −80 °C (years)	Cause of death
PD	1	PR, WB	5	-	73	M	19	5:00	1217	Jan 2016	May 2021	Jun 2021	5.5	Acute respiratory failure secondary to aspiration
	2	PR, WB	5	II	87	M	16	15:15	1370	Dec 2011	Jul 2018	Jun 2021	9.5	Multi-organic failure
	3	PR, WB	4	I-II	83	M	23	11:00	1430	Dec 2014	Jul 2018	Jun 2021	7.5	Acute respiratory failure. Retention stroke.
	4	PR, WB	5	II	62	M	12	13:30	1355	Jun 2013	Jul 2018	Jun 2021	8.0	Cardiorespiratory arrest
	5	PR, WB	5	II	81	M	n.a.	7:20	1402	Dec 2012	Jul 2018	Jun 2021	8.5	Acute cardiorespiratory failure
	6	PR, WB	5	II	75	F	12	3:45	1095	Mar 2013	Jul 2018	Jun 2021	8.3	Multi-organic failure
	7	PR	4	II	77	M	7	12:00	1310	Feb 2011	Jul 2018	Jun 2021	10.3	Multi-organic failure
	8	PR, WB	5	II	78	M	9	5:15	1210	Jun 2012	Mar 2015	Jun 2021	9.0	Respiratory infection (Bronchoaspiration)
NPD	9	PR	-	-	71	F	-	7:08	975	Apr 2019	Dec 2019	Jun 2021	2.2	Cardiorespiratory arrest
	10	PR, WB	-	-	68	M	-	4:00	1220	Mar 2019	Dec 2019	Jun 2021	2.3	Cardiorespiratory arrest
	11	PR	-	-	68	M	-	4:10	1350	Jun 2018	Dec 2019	Jun 2021	3.0	Sepsis
	12	PR, WB	-	-	77	M	-	10:31	1300	Jan 2015	Dec 2019	Jun 2021	6.5	Bronchoaspiration
	13	PR, WB	-	-	72	M	-	9:00	1407	Feb 2013	May 2021	Jun 2021	8.3	n.a.
	14	PR, WB	-	-	81	M	-	5:00	1390	Feb 2013	May 2021	Jun 2021	8.3	Respiratory pathology
	15	PR, WB	-	-	72	M	-	2:55	1340	Sep 2017	May 2021	Jun 2021	3.8	Systemic vascular pathology
	16	PR, WB	-	-	68	F	-	16:30	1076	Feb 2018	May 2021	Jun 2021	3.3	Refractory asystole

Abbreviations: F, female; g, grams; M, male; n.a., not available; NPD, non-Parkinson’s disease; PD, Parkinson’s disease; PMD, post-mortem delayed; PR, proteomic; WB, western blot.

**Table 2:** Demographic, clinicopathological features, and assay of the cases used for stereological analysis

Diagnosis	Case	Assay	Braak syn stage	Braak tau stage	Age (years)	Sex	PMD (hh:mm)	Autopsy brain weight (g)	Duration of PD (years)	Cause of death
PD	17	IH	6	II	75	M	3:00	1350	8	Cardiorespiratory arrest
	18	IH	6	-	73	M	1:00	1450	1,5	Cardiorespiratory arrest
	19	IH	5	III	82	F	2:00	1300	22	Cardiorespiratory arrest
	20	IH	6	0	65	M	n.a.	1305	9	n.a.
	21	IH	6	III	80	M	5:00	1062	n.a.	Bronchial aspiration pneumonia
	22	IH	5	I	80	M	6:00	1231	n.a.	Nosocomial pneumonia
	23	IH	5	0	75	M	5:00	1380	2	n.a.
	24	IH	6	0	68	F	7:00	1132	n.a.	Aspiration pneumonia
	25	IH	5	II	79	F	6:00	1210	9	Respiratory insufficiency
	26	IH	4	I	84	M	5:00	1180	n.a.	Respiratory sepsis
Control (NPD)	27	IH	-	I	63	M	2:00	1400	-	Cardiorespiratory arrest
	28	IH	-	-	62	F	2:00	1050	-	Cardiorespiratory arrest. Multi-organ failure
	29	IH	-	0	58	F	5:00	944	-	Pneumonia
	30	IH	-	0	43	M	5:00	1412	-	Septic shock secondary to pneumonia
	31	IH	-	-	84	M	3:00	1400	-	Cardiac arrest
	32	IH	-	I	56	M	19:00	1400	-	Cardiorespiratory arrest
	33	IH	-	I	74	M	7:00	1336	-	Tumor of unknown origin
	34	IH	-	-	53	M	5:00	1300	-	Cardiorespiratory arrest
	35	IH	-	II	78	M	4:00	1100	-	Respiratory insufficiency
	36	IH	-	II	88	M	3:00	1285	-	n.a.

Abbreviations: F, female; g, grams; IH, immunohistochemistry; M, male; n.a., not available; NPD, non-Parkinson’s disease; PD, Parkinson’s disease; PMD, post-mortem delayed.

The stereology data were analyzed by Stereo Investigator (https://www.mbfbioscience.com/products/stereo-investigator) software, including the area fraction fractionator (fraction of area occupied by α-syn), optical fractionator (density of neurons, microglia, and astroglia), and Cavalieri method (AC and nuclei volume). Comprehensive information on criteria sample selection, processing, analysis, and statistics are provided in the corresponding figures, tables, and main text.

#### Human Postmortem Brain Tissue

Human samples and data were provided by the National Network of Biobanks of Spain, namely, the *Institut d’Investigacions Biomèdiques August Pi i Sunyer*, *Biobanco en Red de la Región de Murcia*, *Biobanco de Tejidos de la Fundación CIEN*, *Biobanco del Principado de Asturias*, and *Biobanco Navarrabiomed* (registration numbers: B.0000575, B.0000859, B.0000741, B.0000827, and B.0000735, respectively).

Samples were selected based on these criteria. Cases neuropathologically diagnosed with PD at Braak α-syn stage 4 to 6 were included in the PD group, while those with no clinical or neuropathological evidence of PD were grouped as NPD. Cases with other neurodegenerative disorders or pathologies that could interfere with the goal of the study, such as small vessel disease, were excluded. However, cases with early-stage Alzheimer's disease (Braak tau) were included (Tables 1 and 2). Only cases that were matched in age and brain weight were chosen, preventing these factors from influencing the results. Therefore, there were no statistically significant differences in age and brain weight between the PD and NPD groups in either the stereological (age: PD 76 ± 2; NPD 66 ± 5) (brain weight: PD 1260 ± 119.0; NPD 1263 ± 169.8) or the proteomic analysis (age: PD 70 ± 8; NPD 72 ± 5) (brain weight: PD 1299 ± 114.8; NPD 1257 ± 156.4). Finally, the distance to bregma was established to range from 4.0 to 10.7 mm for all AC specimens. All potential cases that fit the predetermined criteria were included in the investigation. However, the number of samples that could be collected from tissue biobanks limited the sample size.

### Proteomic Analysis

#### Sample Preparation

From the autopsy, frozen unfixed samples were stored at −80 °C for a mean of 6 ± 3 years (Table 1) and homogenized following the procedures previously described (33, 34, 35). Briefly, tissue was homogenized in 0.4 ml of radioimmunoprecipitation assay buffer containing a protease inhibitor cocktail (Sigma‒Aldrich). The homogenates were agitated (4 °C, 2 h) and centrifuged (4 °C, 12,000*g*, 5 min), and the supernatant was collected.

Samples were precipitated using methanol/chloroform, and the pellet was dissolved in 100 μl of RapiGest SF Surfactant (Waters). Total protein was measured using the Qubit Protein Assay Kit (Thermo Fisher Scientific), and 25 μg of protein was digested using the iST kit (PreOmics). Peptides were diluted using LC‒MS H_2_O 0.1% (v/v) formic acid to 10 ng/μl. Two hundred nanograms of peptides were loaded onto Evotips (Evosep) for purification. Pierce HeLa tryptic Digest Standard (Thermo Fisher Scientific) was also loaded for quality control.

#### Liquid Chromatography-Tandem Mass Spectrometry

Liquid Chromatography-Tandem Mass Spectrometry was carried out using an Evosep One LC system (Evosep) coupled to a TIMS Q-TOF instrument (timsTOF Pro, Bruker Daltonics) *via* a nanoelectrospray ion source (Captive Spray Source, Bruker Daltonics). Samples were separated with the predefined 60 sample per day method (21 min gradient time, 200 ng peptides) on a reverse-phase analytical column (8 cm × 150 μm, C18 1.5 μm (EV1109)) heated to 40 °C. The mobile phases were H_2_O and acetonitrile buffered with 0.1% formic acid (LC‒MS grade, Thermo Fisher Scientific).

To build the MS/MS spectral libraries, a pool of equal mixtures of the original samples were analyzed using data-dependent acquisition-parallel accumulation serial fragmentation (dda-PASEF) (identified proteins in the spectral library are listed in Supplementary Data 1*A*), and the individual samples were analyzed using dia-PASEF. The acquisition modes recorded spectra from 475 to 1000 m/z. Ion mobility resolution was calibrated with three Agilent ESI-L Tuning Mix ions (mass spectra, ion mobility: 622.0289 m/z, 0.9848 V cm^−2^; 922.0097 m/z, 1.1895 V cm^−2^; 1221.9906 m/z, 1.3820 V cm^−2^) and set to 0.85 to 1.30 V cm^−2^. dda-PASEF consisted of four PASEF MS/MS. The accumulation of ions and the ramp times parallel to the scans were 100 ms, with a total cycle time of 0.53 s. Any individually charged ions were excluded from MS/MS analysis. Moreover, precursors that reached the target value of 20,000 were excluded for 0.4 min. A Q window of ±2 Da for m/z <700 and ±3 Da for m/z >800 was used for isolating precursors. The 12 most intense ions were fragmented. For dia-PASEF, 21 nonoverlapping windows of 25 Da were set, resulting in a total cycle time of 0.95 s. The collision energy decreased linearly from 45 eV at an ion mobility of 1.30 Vs cm^−2^ to 27 eV at 0.85 Vs cm^−2^ in both the dda-PASEF and dia-PASEF methods.

#### Protein Identification

FragPipe (17.1), which includes MSFragger (3.4) and EasyPQP (0.1.25) components, was used to build spectral libraries. Peptide identification was performed using MSFragger. Databases of *Homo sapiens* protein sequences (UP000005640) from UniProt (reviewed sequences only; Apr 2021) and common contaminating proteins, which contained 20,382 total sequences, were used. Inverted protein sequences were added to the original databases. The initial mass tolerance was set at 20 ppm for precursor and fragment ions. Trypsin was set as described above with a maximum of two missed cleavages. Methionine oxidation and N-terminal acetylation were established as variable modifications, and carbamidomethylation was established as a fixed modification. A peptide length range of 7 to 50 amino acids and a peptide mass range of 500 to 5000 Da were set. The maximum number of variable modifications per peptide was fixed at 3. PeptideProphet was used to calculate the probability of correct identification of peptides for spectrum matching and to assemble peptides into proteins. Philosopher Filter was used to assign each identified peptide as a razor peptide to a single protein or protein group that had the greatest peptide evidence. The false discovery rate (FDR) was set to 1% for peptide spectrum match or ion/peptide and protein identification. EasyPQP was used for aligning peptides to a common indexed retention time scale and peptide ion mobility to that from one of the reference runs automatically selected. The final spectral library was filtered at a 1% FDR at the peptide and protein levels.

DIA-NN 1.8 (https://github.com/vdemichev/DiaNN/releases/tag/1.8) was used for dia-PASEF analysis and operated with maximum mass tolerances set to 15 ppm. The samples were analyzed with run-to-run pairing (match between ranks) enabled. Protein inference in DIA-NN was configured to use the assembled proteins in the spectral library. “Protein.Group” column in the DIA-NN report was used to identify the protein group, and PG.MaxLFQ label-free quantification was used to obtain the normalized amount. Tables generated by Philosopher were used to identify proteotypic peptides. The quantification mode was set to "Any LC (high precision)". All other settings were left as default. The DIA-NN output was filtered at a q value <1% for precursors and proteins.

The FDR validation was filtered to include only unmodified peptides or peptides with carbamidomethylated cysteines, oxidized methionines, or excised N-termini of methionines. The library was screened for precursors/proteins with a 2 to 4 charge range and a 100.0 to 1700.0 m/z mass range.

As mentioned above, dia-PASEF proteomics analysis was carried out at the *Instituto Maimonides de Investigación Biomédica de Córdoba* IMIBIC Proteomic Facility. The mass spectrometry proteomics data have been deposited to the ProteomeXchange Consortium (http://proteomecentral.proteomexchange.org) *via* the PRIDE partner repository with the dataset identifier PXD043503 (Username: reviewer_pxd043503@ebi.ac.uk; Password: eHWcHRb6).

#### Data analysis

Perseus (1.6.15.0) was used to analyze the identified proteins. After log2 transformation, data were normalized using the width adjustment method. Proteins with one razor peptide were removed, and missing values were imputed (75% minimum valid in each group). An unpaired two-tailed *t* test (*p*-value <0.05) was employed to estimate significant differences. Proteins with a fold change ≥1.3 and ≤0.77 were considered upregulated and downregulated, respectively. Proteins that interact with α-syn were obtained by the BioGRID (https://thebiogrid.org) (4.4.211). The Human Body Fluid Proteome (https://bmbl.bmi.osumc.edu/HBFP) database was used to test whether DEPs that are α-syn interactors were found in human body fluids. Lists of proteins enriched in neurons, microglia, and astroglia were taken from published experimental data (36). SynGO (https://syngoportal.org) (20210225) was employed to determine which DEPs are in the synapse and what role they play in it. IPA (http://www.ingenuity.com) was performed for the analysis of biofunctional pathways and protein‒protein interaction networks of DEPs and MAP2 (neuronal protein), AIF1 (microglial protein), GFAP (astroglial protein), and SNCA (α-syn protein).

#### Immunofluorescence and Western Blotting for Proteomic Validation

Immunofluorescence and Western blot analysis were carried out as previously published (33, 34, 35).

For qualitative validation, an immunofluorescence analysis was performed (Table 2). Tissue antigenicity was unmasked by boiling the sections in citrate buffer under pressure. H_2_O_2_ (1%) inactivated the activity of endogenous peroxidases. Sections were incubated at 4 °C with shaking with the primary antibody and fluorescent secondary antibody at RT (Table 3). Sections were counterstained with 0.01% 4′,6-diamidino-2-phenylindole and coated with PVA-DABCO (Sigma‒Aldrich) (34). Images were captured with a Zeiss LSM 800 confocal microscope coupled to Zen 2.3 (https://www.zeiss.com/microscopy/es/productos/software/zeiss-zen.html) software.

**Table 3:** Primary and secondary antibodies used

Assay	Antigen	Company	Catalog n°	Host	Blocking buffer	Incubation	Secondary antibody
IMMUNOHISTOCHEMISTRY	α-Synuclein	Novocastra	NCL-L-ASYN	Mouse monoclonal	-	1:20 in PBS +0.3% TX-100 (4 °C, 72 h)	1:200 Biotinylated horse anti-mouse IgG (H+L) Vector laboratories in PBS + 0.03% TX-100 (2 h)
	MAP2	Thermo Fisher Scientific	13-1500	Mouse monoclonal	PBS +0.4% TX-100 + 3% NHS (1 h)	1:500 in BB (4 °C, ON)	1:200 Biotinylated horse anti-mouse IgG (H+L) Vector laboratories in BB (2 h)
	Iba-1	Fujifilm WAKO	019-19741	Rabbit polyclonal	PBS +0.1% TX-100 (2 h)	1:2,00 in BB (4 °C, ON)	1:200 Biotinylated horse anti-rabbit IgG (H+L) Vector laboratories in BB (2 h)
	GFAP	Dako	Z0334	Rabbit polyclonal	PBS +0.1% TX-100 + 10% NHS (2 h)	1:10,000 in BB (4 °C, ON)	1:200 Biotinylated horse anti-rabbit IgG (H+L) Vector laboratories in BB (2 h)
IMMUNOFLUORESCENCE	MAP2	Thermo Fisher Scientific	13-1500	Mouse monoclonal	TBS +0.3% TX-100 + 10% NDS (30 min)	1:100 in TBS +0.03% TX-100+ 10% NDS (4 °C, 72 h)	1:200 Alexa Fluor 488 donkey anti-mouse IgG (H+L) Invitrogen in TTBS + 0.3 TX-100 (2 h)
	eEF1A1	Abcam	ab140632	Rabbit monoclonal	TBS +0.3% TX-100 + 10% NDS (30 min)	1:150 in TBS +0.03% TX-100 + 10% NDS (4 °C, 72 h)	1:200 Alexa Fluor 568 donkey anti-rabbit IgG (H+L) Invitrogen in TTBS + 0.3 TX-100 (2 h)
	NPTN	Abcam	ab272652	Rabbit polyclonal	TBS +0.3% TX-100 + 10% NDS (30 min)	1:50 in TBS +0.03% TX-100 + 10% NDS (4 °C, 72 h)	1:200 Alexa Fluor 568 donkey anti-rabbit IgG (H+L) Invitrogen in TTBS + 0.3 TX-100
	YWAHAH	Abcam	ab206292	Rabbit monoclonal	TBS +0.3% TX-100 + 10% NDS (30 min)	1:50 in TBS +0.03% TX-100 + 10% NDS (4 °C, 72 h)	1:200 Alexa Fluor 568 donkey anti-rabbit IgG (H+L) Invitrogen in TTBS + 0.3 TX-100 (2 h)
	Milli-Mark Pan Neuronal Marker (PAN)	Merck	MAB2300	Mouse monoclonal	TBS +0.3% TX-100 + 10% NDS (30 min)	1:100 in +0.03% TX-100 (4 °C, 72 h)	1:200 Alexa Fluor 488 donkey anti-mouse IgG (H+L) Invitrogen in TTBS + 0.3 TX-100 (2 h)
	Myelin PLP	Abcam	ab254363	Rabbit monoclonal	TBS +0.3% TX-100 + 10% NDS (30 min)	1:1500 in + 0.03% TX-100 (4 °C, 72 h)	1:200 Alexa Fluor 568 donkey anti-rabbit IgG (H+L) Invitrogen in TTBS + 0.3 TX-100 (2 h)
	GFAP	Abcam	ab53554	Goat polyclonal	TBS +0.3% TX-100 (30 min)	1:500 in +0.03% TX-100 (4 °C, 72 h)	1:200 Alexa Fluor 647 donkey anti-goat IgG (H+L) Invitrogen in TTBS + 0.3 TX-100 (2 h)
	Orosomucoid-2 (ORM2)	Abcam	ab231906	Rabbit polyclonal	TBS +0.3% TX-100 (30 min)	1:50 in +0.03% TX-100 (4 °C, 72 h)	1:200 Alexa Fluor 568 donkey anti-rabbit IgG (H+L) Invitrogen in TTBS + 0.3 TX-100 (2 h)
	Iba-1	Fujifilm WAKO	019-19741	Rabbit polyclonal	TBS +0.3% TX-100 (30 min)	1:1000 in TBS +0.03% TX-100+ 10% NDS (4 °C, 72 h)	1:200 Alexa Fluor 568 donkey anti-rabbit IgG (H+L) Invitrogen in TTBS + 0.3 TX-100 (2 h)
	GRIM19	Abcam	ab110240	Mouse monoclonal	TBS +0.3% TX-100 + 10% NDS (30 min)	1:50 in TBS +0.03% TX-100 (4 °C, 72 h)	1:200 Alexa Fluor 568 donkey anti-mouse IgG (H+L) Invitrogen in TTBS + 0.3 TX-100 (2 h)
	GNAI1	Abcam	ab140125	Rabbit monoclonal	TBS +0.3% TX-100 (30 min)	1:50 in TBS +0.03% TX-100 (4 °C, 72 h)	1:200 Alexa Fluor 568 donkey anti-rabbit IgG (H+L) Invitrogen in TTBS + 0.3 TX-100 (2 h)
WESTERN BLOT	GAPDH	Cell Signaling Technology	#2118	Rabbit monoclonal	Skim milk 5% (1 h)	1:2000 in BSA 5% (4 °C, ON)	1:5000 Polyclonal Goat anti-rabbit Immunoglobulins/HRP. Agilent. in TTBS 1X (1 h)
	GNAI1	Abcam	ab140125	Rabbit monoclonal	Skim milk 5% (1 h)	1:1000 in skim milk 0.5% (4 °C, ON)	1:5000 Polyclonal Goat anti-rabbit Immunoglobulins/HRP. Agilent. in skim milk 0.5% (1 h)
	eEF1A1	Abcam	ab140632	Rabbit monoclonal	Skim milk 5% (1 h)	1:2000 in skim milk 0.5% (4 °C, ON)	1:5000 Polyclonal Goat anti-rabbit Immunoglobulins/HRP. Agilent. in skim milk 0.5% (1 h)
	Myelin PLP	Abcam	ab254363	Rabbit monoclonal	Skim milk 5% (1 h)	1: 2000 in skim milk 0.5% (4 °C, ON)	1:5000 Polyclonal Goat anti-rabbit Immunoglobulins/HRP. Agilent. in skim milk 0.5% (1 h)
	NPTN	Abcam	ab272652	Rabbit polyclonal	Skim milk 5% (1 h)	1:2500 in skim milk 0.5% (4 °C, ON)	1:5000 Polyclonal Goat anti-rabbit Immunoglobulins/HRP. Agilent. in skim milk 0.5% (1 h)
	YWAHAH	Abcam	ab206292	Rabbit monoclonal	Skim milk 5% (1 h)	1:1000 in skim milk 5% (4 °C, ON)	1:5000 Polyclonal Goat anti-rabbit Immunoglobulins/HRP. Agilent. in skim milk 2.5% (1 h)
	GRIM19	Abcam	ab110240	Mouse monoclonal	Skim milk 5% (1 h)	1:1000 in skim milk 0.5% (4 °C, ON)	1:5000 Goat anti-mouse IgG (H+L) HRP-conjugated. ANASPEC. in skim milk 0.5% (1 h)
	Orosomucoid-2 (ORM2)	Abcam	ab231906	Rabbit polyclonal	Skim milk 5% (1 h)	1:5000 in skim milk 0.5% (4 °C, ON)	1:5000 Polyclonal Goat anti-rabbit Immunoglobulins/HRP. Agilent. in skim milk 0.5% (1 h)

For quantitative validation, Western blot analysis was performed (Table 1). Protein concentration was measured with the bicinchoninic acid protein determination kit (Sigma‒Aldrich) and a Multiskan FC microplate photometer (Thermo Fisher Scientific). Twenty micrograms of protein per sample was loaded onto 10% polyacrylamide gels and separated by SDS‒PAGE. Transfer to polyvinylidene difluoride membranes was performed with a *Trans*-blot Turbo transfer system (Bio-Rad). Membranes were blocked with skim milk and incubated with primary antibodies at 4 °C and with secondary antibodies at RT (Table 3). After incubation with enhanced chemiluminescence reagents (Thermo Fisher Scientific), the band intensities were revealed with SyngeneG:BOX (GeneSys software: https://www.syngene.com/software/genesys-rapid-gel-image-capture/) and analyzed with ImageJ (https://imagej.net/ij/) (33, 35). GraphPad Prism 6.01 (https://www.graphpad.com/) was used for statistical analyses. Outliers identified by the ROUT method were removed. Shapiro–Wilk tests (n < 30) were performed to analyze the normality of the sample (*p*-value >0.05). Statistical comparisons were performed using the unpaired Mann‒Whitney test. Data are presented as the mean ± SD. Significant differences were considered when *p*-value <0.05.

### Stereological Analysis

#### Immunohistochemical Procedures

Tissue processing and immunohistochemical staining were carried out following the procedures previously described in our laboratory (34) (Table 2). Briefly, tissue was postfixed in 4% paraformaldehyde and cryoprotected with 2% dimethyl sulfoxide solution and 10% glycerol and, subsequently, with 2% dimethyl sulfoxide solution and 20% glycerol. Samples were cut with a freezing microtome into 50 μm coronal sections. Sections were collected in series: one was Nissl-stained, and the remaining sections were used for immunohistochemical assays. To unmask epitopes, sections were boiled in citrate buffer under pressure. H_2_O_2_ (1%) inactivated the activity of endogenous peroxidases. Sections were blocked and incubated at 4 °C with shaking with primary antibodies (Table 3) and subsequently with the secondary biotinylated antibody at RT (Table 3). Avidin-biotin complex (ABC standard, Vectastain) was applied and reacted with 0.025% 3,3′-diaminobenzidine and 0.1% H_2_O_2_. Sections were mounted on slides and covered with DPX (Sigma‒Aldrich).

#### Stereological Quantification

Stereo Investigator software (MBF Bioscience coupled to a Zeiss Axio Imager M2 microscope) was used for stereological analyses. Under low magnification, the basolateral (Ba), including basolateral, basomedial, and lateral nuclei; cortical (Co), including anterior and posterior cortical nuclei; and central (Ce) amygdaloid nuclear group limits, were outlined (Plan-Neofluar 1x/0.025, Ref. 420300-9900) (26, 37). Different stereological probes were used to analyze the fraction of area occupied by α-syn, cell population density, and volume.

The area fraction occupied by α-syn was analyzed using the area fraction fractionator method (Plan Apochromat 20x/0.5, Ref. 420650-9901). This analysis estimates the percentage of area occupied by α-syn. The optical fractionator method was applied to analyze the density (estimated population using the mean section thickness/measured volume) of neurons (MAP2), microglia (Iba-1), and astroglia (GFAP) (Plan Apochromatic, 63x/1.4, Oil lens, Ref. 420782-9900). The number of cells counted in the study area was divided by the estimated volume of the region. These quantifications were performed under single-blind conditions. The volume was estimated with a Cavalieri probe using Nissl staining (Plan-Neofluar 1x/0.025, Ref. 420300-9900). More details about these stereological methods were previously described (34, 38).

#### Statistical Analysis

GraphPad Prism (6.01) was used for statistical analyses. Shapiro‒Wilk tests (n < 30) were performed to analyze the normality of the sample (*p*-value > 0.05). Statistical comparisons were performed using unpaired two-tailed t tests to estimate significant differences in age, brain weight, volume, and MAP2, Iba-1, and GFAP densities. The Kruskal‒Wallis test was used to calculate the significance of the area fraction occupied by α-syn. To analyze the correlation between the area fraction occupied by α-syn and the duration of PD, Spearman's test was employed. All data are presented as the mean ± SD. Significant differences were considered when *p*-value <0.05.

## Results

### Proteomic Analysis

**Fig. 1:**
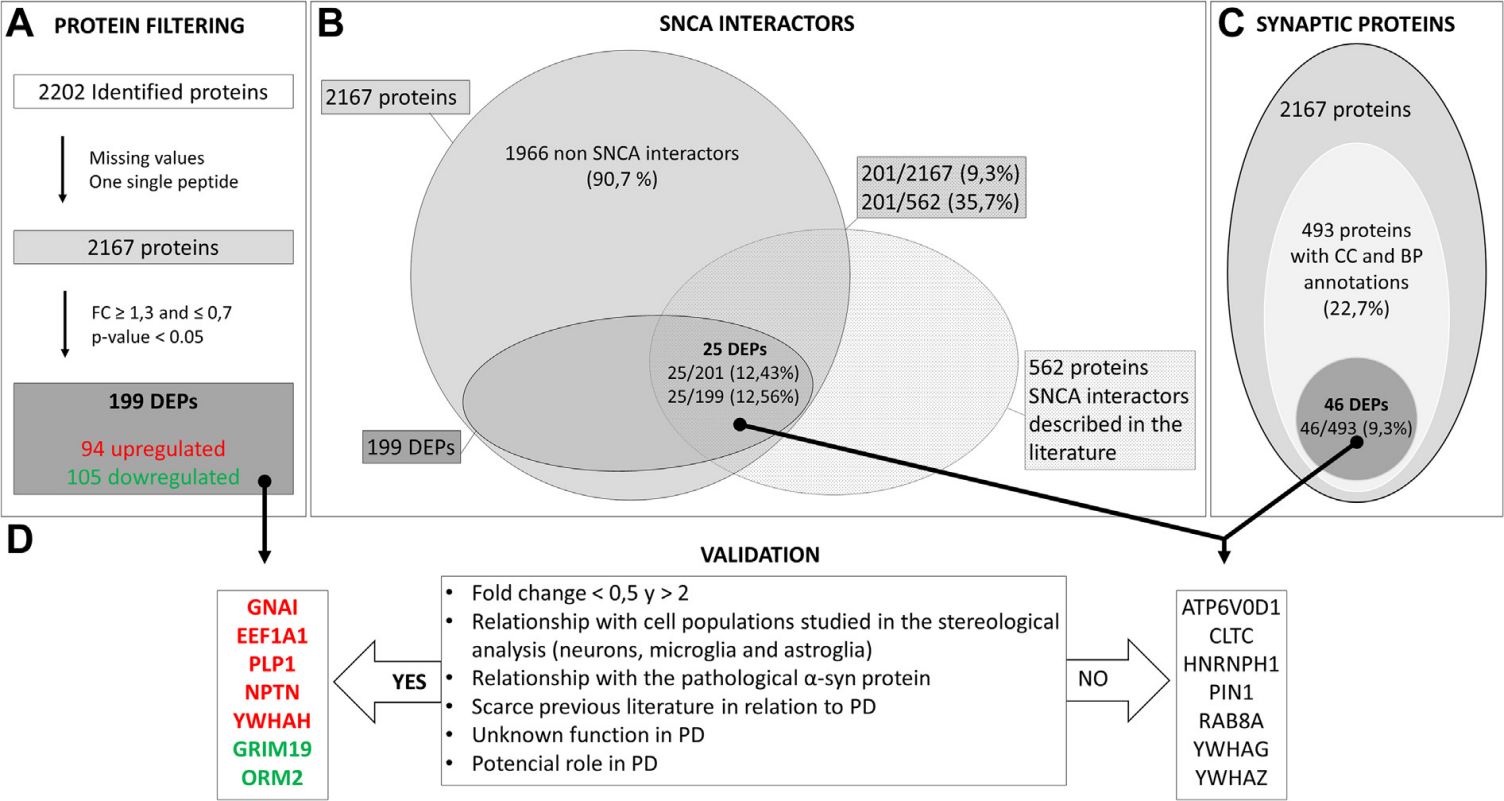
**Summary of proteomic results.** The diagram shows the different bioinformatics analyses carried out, differentially expressed proteins (DEPs) obtained, and those that have passed the criteria established for their validation. *A*, Dia-PASEF proteomic analysis identified 2202 proteins, of which 2167 were quantified. One hundred ninety-nine differentially expressed proteins (94 upregulated and 105 downregulated) were obtained using a *p*-value <0.05 and a fold change ≥1.3 and ≤0.7. *B*, of the total proteins that are composed of the annotated interactome of the SNCA (562 proteins), 35.7% (201/562) were quantified in this study, while 12.43% of the quantified proteins that interact with SNCA are DEPs (25/201). *C*, 22.7% (493/2167) of the quantified proteins comprised synaptic cellular components (CC) and synaptic biological processes (BP), and 9.3% (46/493) of these were DEPs. *D*, five upregulated (*red*) DEPs (GNAI1, EEF1A1, PLP1, NPTN, YWHAY) and two downregulated (*green*) DEPs (GRIM19, ORM2) were selected for the study according to the criteria established for it. The few matching DEPs of the annotated interactome of the SNCA and comprised synaptic CC and synaptic BP did not meet the criteria for selection. dia-PASEF, data-independent acquisition-parallel accumulation serial fragmentation.

**Fig. 2:**
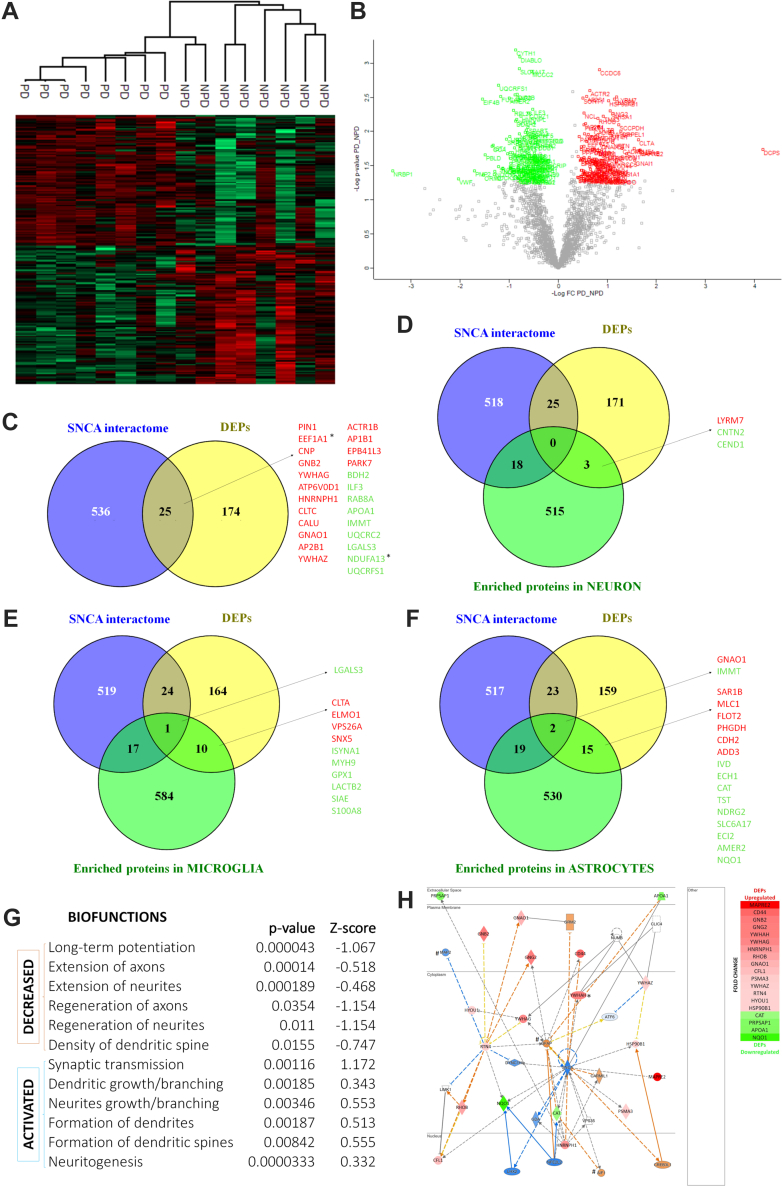
**Proteomic analysis.** The heatmaps of the 212 significant proteins identified (*p*-value <0.05) show two different sample groups: non-Parkinson (NPD) and Parkinson (PD) (*A*). The volcano plot shows 94 upregulated DEPs (in *red*) and 105 downregulated DEPs (in *green*) with a fold change of at least 30% and *p*-value <0.05 (*B*). Venn diagrams (*C*–*F*) showing DEPs that interact with α-synuclein (SNCA) (*C*), DEPs enriched in neurons that interact with SNCA (*D*), DEPs enriched in microglia that interact with SNCA (*E*), and DEPs enriched in astroglia that interact with SNCA (*F*). Biofunctions activated or decreased in relation to synapses in PD (*G*). The protein‒protein interaction network shows the rich interaction of SNCA with DEPs (upregulated in *red* and downregulated in *green*) and with MAP2, AIF1, and GFAP (marked with #). For a complementary legend, see Supplementary Data 2 (*H*). ∗ Some validated proteins (*C*–*F*, *H*). https://qiagen.my.salesforce-sites.com/KnowledgeBase/articles/Knowledge/Legend/p. DEP, differentially expressed protein.

Dia-PASEF proteomic analysis identified 2202 proteins (Supplementary Data 1*B*), of which 2167 proteins with at least two unique peptides were quantified (Supplementary Data 1*C*). To facilitate the interpretation and follow-up of the proteomic results, the reader is encouraged to view Figure 1 in conjunction with Figure 2. Statistically significant proteins in both the PD and NPD groups showed different expression patterns (Fig. 2*A*, Supplementary Data 1*D*). A total of 199 proteins were DEPs in the ACs of humans with PD (94 upregulated and 105 downregulated) (Figs. 1*A* and 2*B*, Supplementary Data 1*E*). Of the DEPs, 25 (16 upregulated and nine downregulated) were identified as SNCA interactors (Figs. 1B and 2*C*, Supplementary Data 1*F*) and all were found in biofluids such as plasma, serum, cerebrospinal fluid, saliva, urine, etc. Of the total proteins that are composed of the annotated interactome of the SNCA (562 proteins), 35.7% (201 proteins) were quantified in this study. While 12.43% of the quantified proteins that interact with SNCA are DEPs (25 of the 201), only 8.85% of the noninteractome-quantified proteins are differentially regulated (174 of the 1966) (Fig. 1*B*). Regarding cellular populations, Venn diagram comparisons of the list of 199 DEPs with the published lists of proteins enriched in neurons, microglia, and astrocytes (36) revealed that three DEPs—one upregulated and two downregulated—were enriched in neurons (Fig. 2*D*), 11 (four upregulated and seven downregulated) in microglia (Fig. 2*E*), and 17 (seven upregulated and 10 downregulated) in astrocytes (Fig. 2*F*). Moreover, one of these typical microglial proteins and two typical astrocytic proteins are part of the α-syn interactome (Fig. 2, *E* and *F*). Studying the role of synaptic proteins in PD is crucial given the synaptic location of α-syn. In SynGO, 22.75% (493/2167) of the proteins identified in the proteomic study had annotations for cellular components and biological processes. Of them, 9.33% (46/493) were differentially expressed in the AC in PD (Fig. 1*C*, Supplementary Data 1*G*). In addition, upregulated and downregulated DEPs activated or decreased biofunctions in relation to synapses (Fig. 2*G*, Supplementary Data 1*H*). The protein‒protein interaction network showed that the pathological protein SNCA has several direct and indirect interactions with many of the DEPs and with the neuronal (MAP2), microglial (AIF1, synonymous with Iba-1), and astroglial (GFAP) proteins analyzed by stereology (Fig. 2*H*, Supplementary Data 2).

#### Protein Validation

**Fig. 3:**
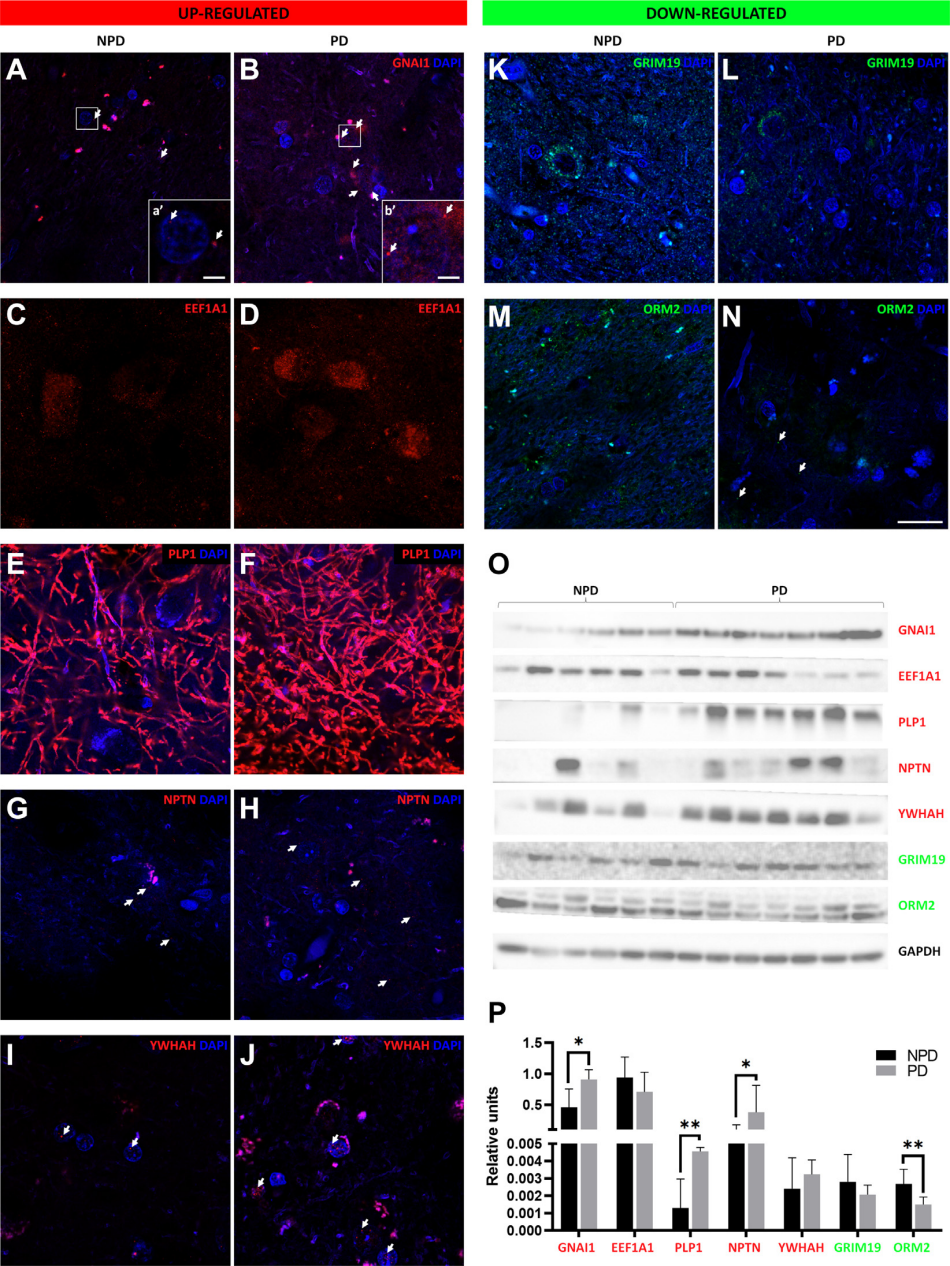
**Proteomic validation.** Coronal sections of the human amygdaloid complex immunofluorescently stained for qualitative validation (*A*–*N*) and quantitative validation by Western blot of differentially expressed proteins (*O* and *P*). The upregulated differentially expressed proteins (GNAI1, EEFIA1, PLP1, NPTN, and YWHAH) are shown in *red* and the downregulated proteins (GRIM19 and ORM2) are shown in *green*. *Arrows point* to markers. Scale bars represent 20 μm (*A*–*N*) and 2.5 μm (a’, b’). Significance ∗*p*-value < 0.05, ∗∗*p*-value < 0.01.

Five upregulated DEPs—guanine nucleotide-binding protein G(i) subunit alpha-1 (GNAI1), elongation factor 1-alpha 1 (EEF1A1), myelin proteolipid protein (PLP1), neuroplastin (NPTN), and 14-3-3 protein eta (YWHAH)—and two downregulated DEPs—NADH dehydrogenase [ubiquinone] 1 alpha subcomplex subunit 13 or gene associated with retinoic and interferon-induced mortality 19 protein (NDUFA13, GRIM19, respectively) and orosomucoid-2 (ORM2)—were qualitatively (Figs. 1*D* and 3, *A*–*N*) and quantitatively validated (Figs. 1*D* and 3, *O* and *P*). The validated DEPs met the following criteria: fold change <0.5 or >2, relationship of interest with the cell populations studied in the stereological analysis (neurons, microglia, and astroglia) or with the pathological α-syn protein, previously poorly studied, unknown function and potential role in relation to PD (Fig. 1*D*). Qualitatively, what was observed by immunofluorescence coincided with the results obtained from the proteomic analysis. Quantitative Western blot analysis indicated significant upregulation of GNAI1, PLP1, and NPTN in PD and significant downregulation of ORM2 in PD. EEF1A1, YWHAH, and GRIM19 did not show significant differences between NPD and PD.

**Fig. 4:**
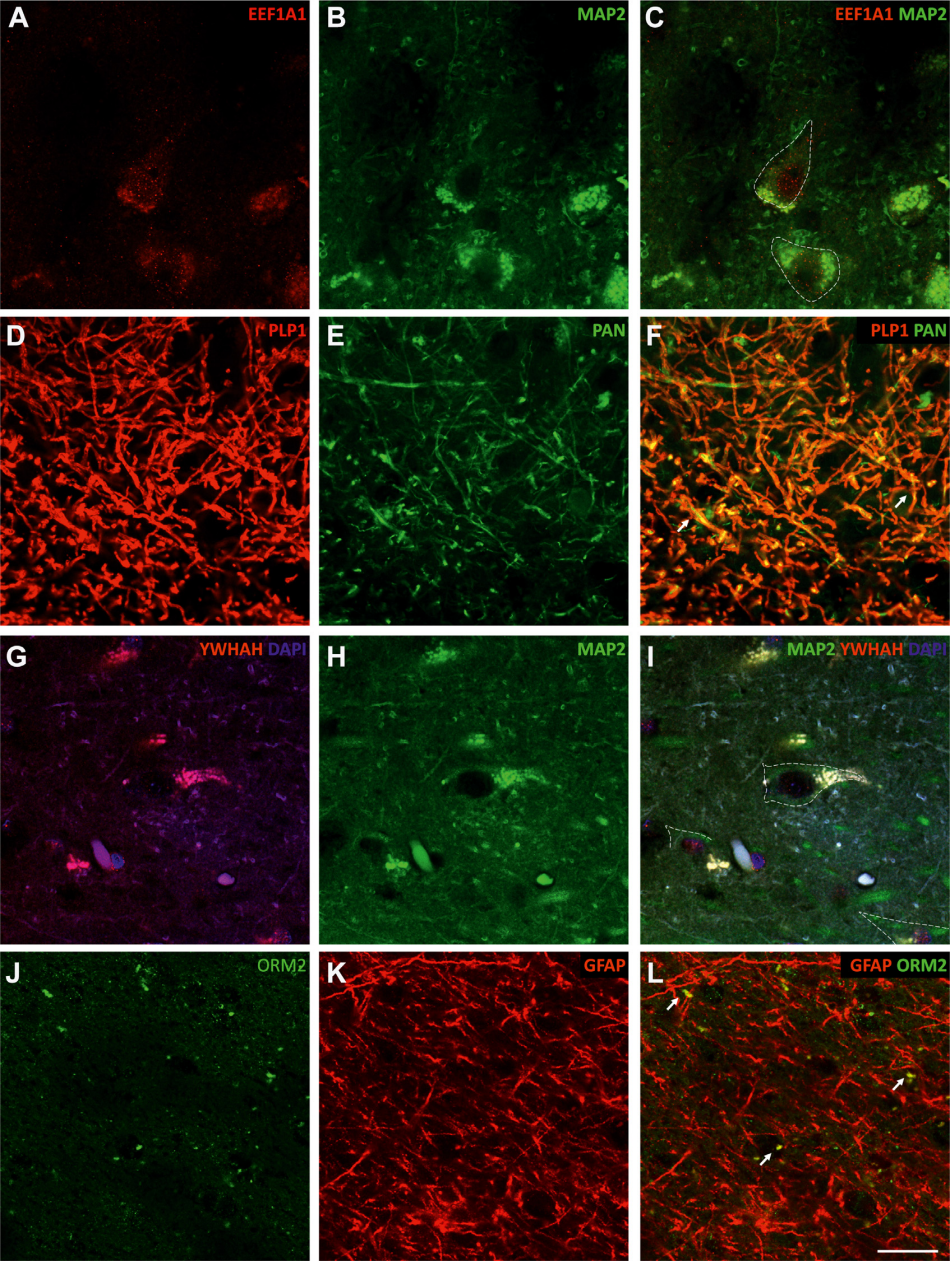
**Localization of differentially expressed proteins in stereological cell types.** EEF1A1 (*A* and *C*) labels neurons as does the neuronal-specific antibody MAP2 (*B* and *C*). PLP1 (*D* and *F*) colocalizes with the axonal marker PAN (*E* and *F*). YWHAH (*G* and *I*) is especially localized in the nucleus of MAP2-tagged neurons (*H* and *I*). ORM2 (*J* and *L*) barely colocalizes with GFAP (*K* and *L*). *Dashed lines* delineate neuronal somas. *Arrows point* to colocalizations. Scale bar represents 20 μm.

In addition, the potential localization of DEPs in neurons, microglia, and astroglia was analyzed. EEF1A1 labeled neurons immunoreactive for MAP2 (Fig. 4, *A*–*C*). PLP1 labeled the immunoreactive axons for PAN (Fig. 4, *D*–*F*). YWHAH localized especially to the nucleus of neurons immunospecific for MAP2 (Fig. 4, *G*–*I*). ORM2 rarely colocalized with GFAP-labeled astroglial cells (Fig. 4, *J*–*L*). NPTN and GNAI1 were distributed homogeneously throughout the tissue without localization preferences (data not shown). GRIM19 could not be studied together with other antibodies.

Alterations in other DEPs—peptidyl-prolyl *cis*-*trans* isomerase NIMA-interacting 1 (PIN1), CD44 antigen (CD44), and putative tyrosine-protein phosphatase auxilin (DNAJC6)—coincide with what has been reported in the literature in relation to PD, which makes our analysis reliable (Supplementary Data 1*E*). In addition, other DEPs coincidence with synapses and SNCA interactors, including V-type proton ATPase subunit d1 (ATP6V0D1), clathrin heavy chain 1 (CLTC), heterogeneous nuclear ribonucleoprotein H (HNRNPH1), peptidyl-prolyl *cis*-*trans* isomerase NIMA-interacting 1 (PIN1), Ras-related protein Rab-8A (RAB8A), 14-3-3 protein gamma (YWHAG), and 14-3-3 protein zeta/delta (YWHAZ). Nevertheless, these proteins did not meet the established criteria for validation, as they have been widely reported in the literature, and their main role in PD is known (Fig. 1*D*).

### Stereological Analysis

#### α-Synuclein Area Fraction

**Fig. 5:**
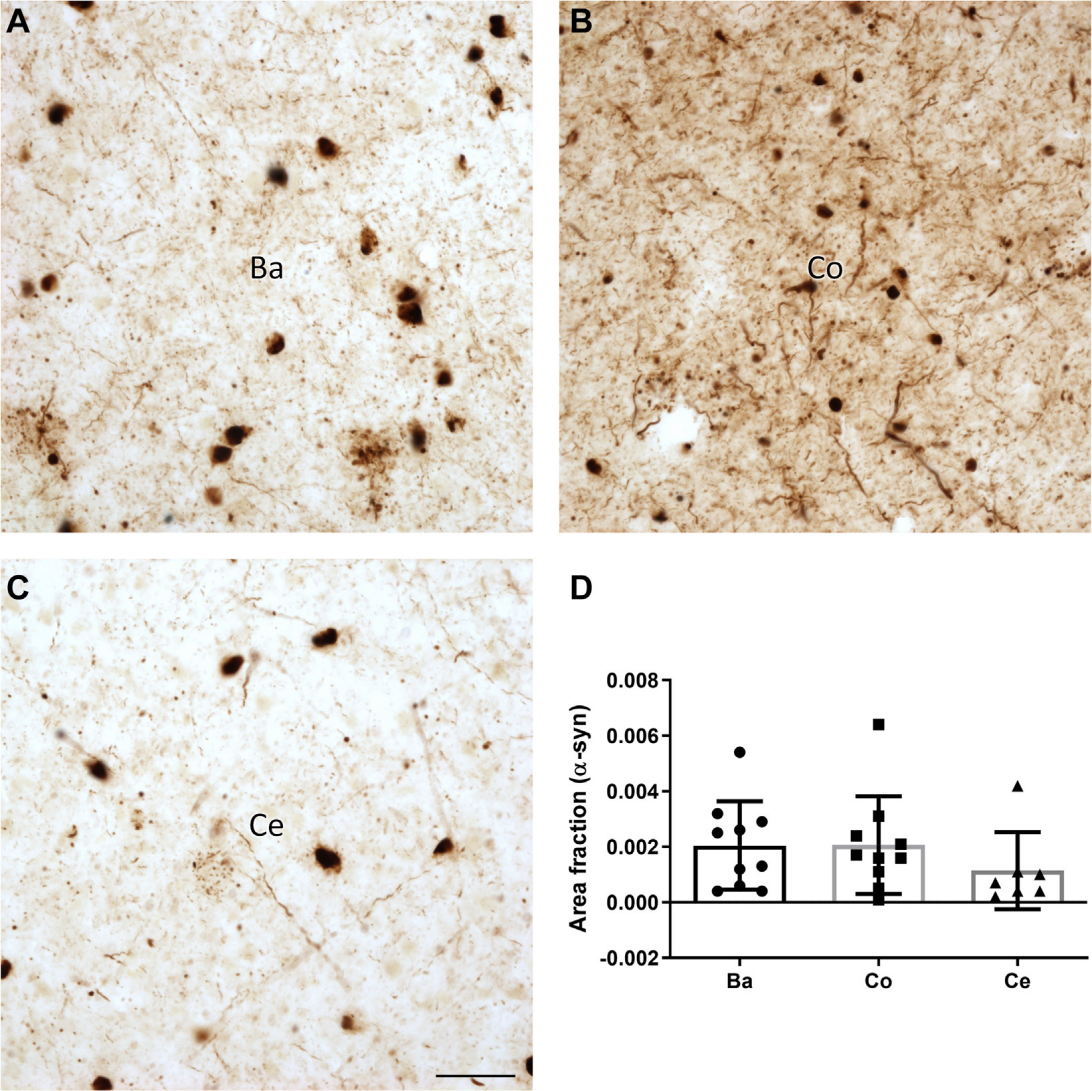
**Analysis of the area fraction occupied by α-synuclein in the human amygdaloid complex.** Basolateral (Ba) (*A*), cortical (Co) (*B*), and central (Ce) (*C*) nuclear groups stained with α-syn. The mean ± SD of the α-synucleinopathy fraction of the area of Parkinson's disease cases remained unchanged in all nuclear groups studied (*D*). Scale bar represents 50 μm. α-syn, α-synuclein.

The area fraction occupied by α-syn in the Ba, Co, and Ce nuclei of the AC did not show significant differences in the PD group (Fig. 5, see Supplemental Table S1 for stereological data).

**Supplemental Figure S1:**
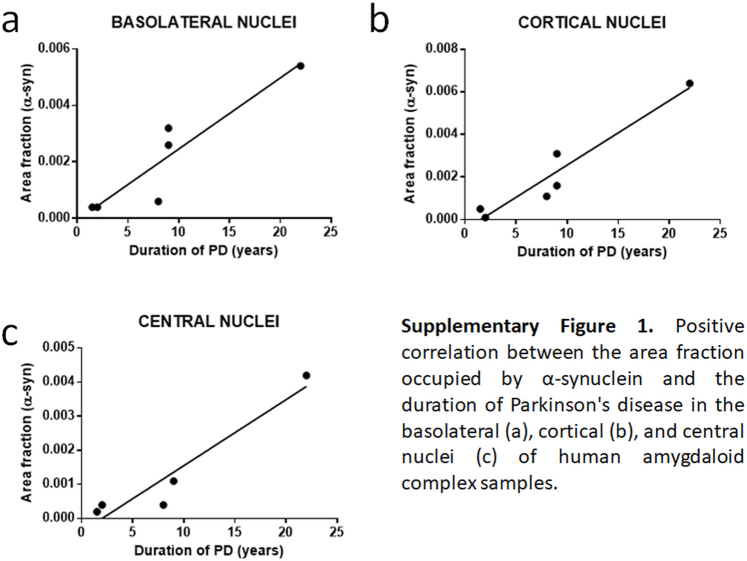


Interestingly, the duration of PD correlates positively with the values of the area fraction occupied by α-syn in each nucleus of the AC (Supplemental Fig. S1).

#### Cell Populations and Volume Estimation

**Fig. 6:**
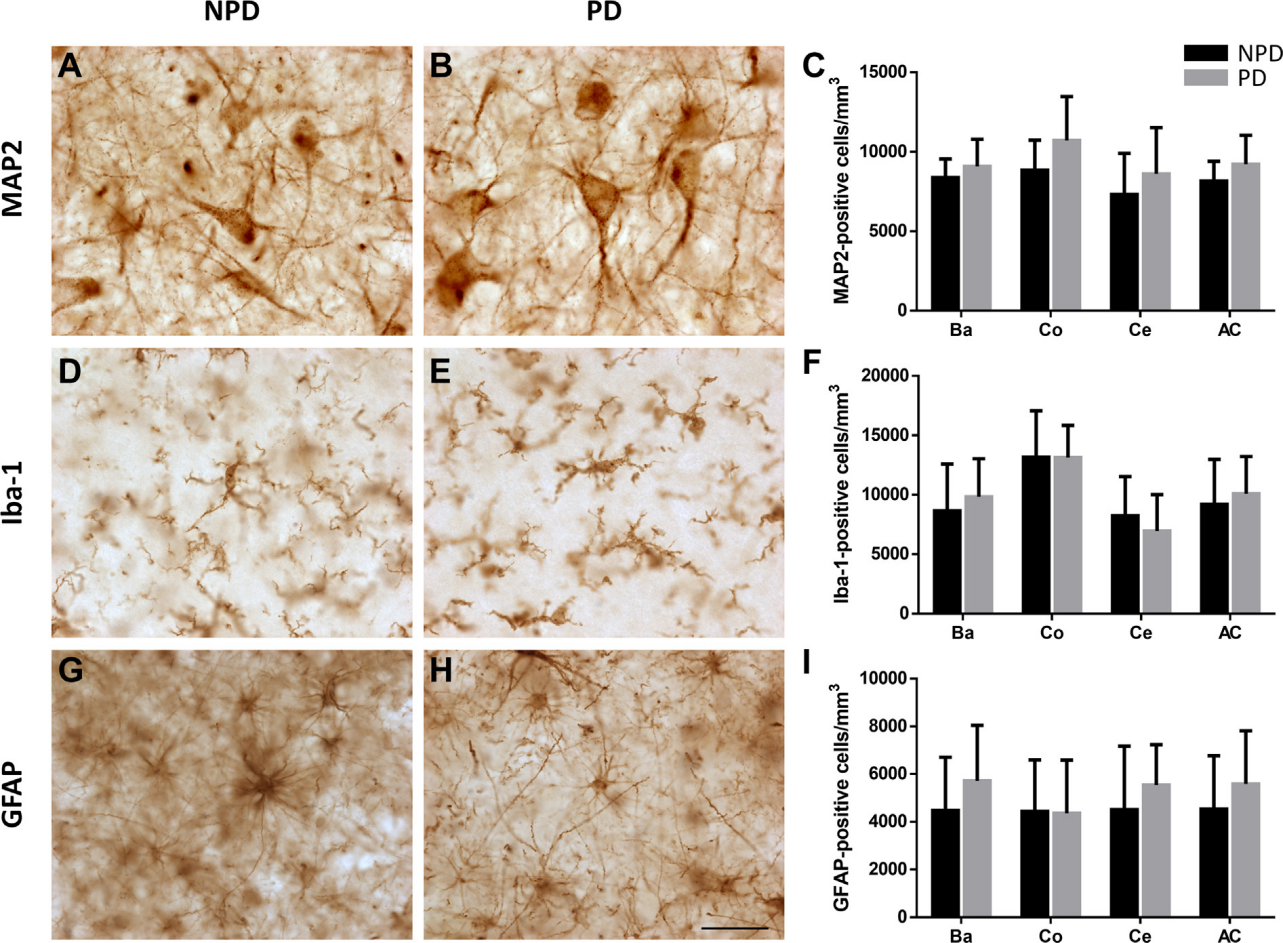
**Quantification of the neuronal, microglial, and astroglial density of the human amygdaloid complex.** Neuronal, microglial, and astroglial populations stained with MAP2 (*A* and *B*), Iba-1 (*D* and *E*), and GFAP (*G* and *H*), respectively, in non-Parkinson's (NPD) and Parkinson's disease (PD) cases. The mean ± SD of MAP2- (*C*), Iba-1- (*F*), and GFAP-positive cell density (*I*) of the amygdaloid complex (AC) and its nuclear groups basolateral (Ba), cortical (Co), and central (Ce) did not differ between study groups. Scale bar represents 25 μm.

**Fig. 7:**
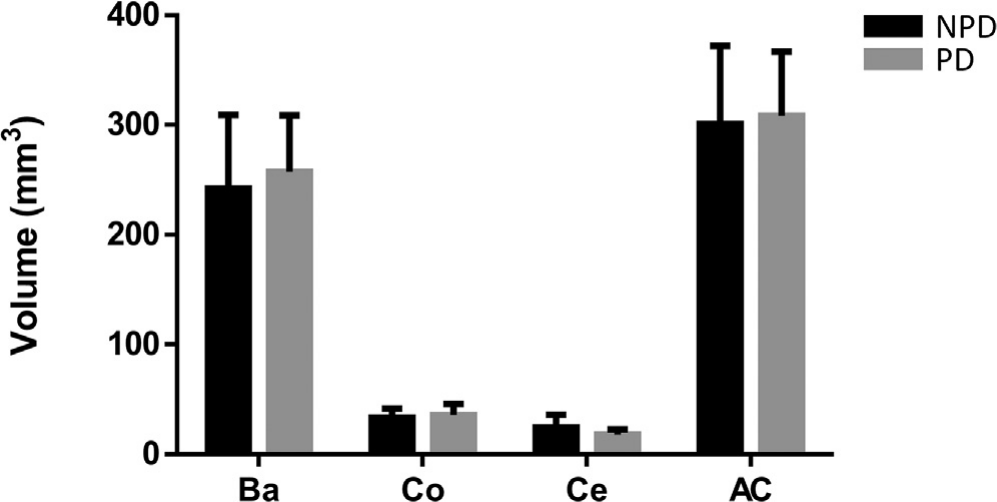
**Amygdaloid complex and nuclei volume.** The mean ± SD of the volume does not differ between non-Parkinson’s (NPD) and Parkinson’s disease (PD) samples (c). Scale bar represents 100 μm.

MAP2-, Iba-1-, and GFAP-positive cell density remained stable in the AC and the Ba, Co, and Ce nuclei in the PD and NPD groups (Fig. 6, see Supplemental Tables S2–S4 for stereological data). The volume of the total AC and its nuclei remained unchanged between the PD and NPD groups (Fig. 7, see Supplemental Table S5 for stereological data).

## Discussion

As stated, α-syn is the proteinopathy associated with PD constituting Lewy pathology (10), allowing neuropathological staging (4). Lewy pathology progresses in the brain in a prion-like manner (39) involving many prodromal structures until stage 3, when the substantia nigra, which provokes the motor diagnosed symptoms, and the AC are involved (10). The AC is an essential hub in synucleinopathy progression (8); therefore, proteomic analysis has focused on α-syn interactors and synaptic involvement. In parallel, α-syn distribution, neural (40) and glial (41) involvement, and AC volume have been discussed in previous controversial literature.

Proteomic alterations could provide clues as to how the biofunctions of cell populations are altered in PD. To date, most proteomic studies in PD have focused on the substantia nigra (23). However, our study is the first to analyze the proteome of the AC of patients with PD and revealed significant damage at the synaptic level (Figs. 1 and 2). α-Syn is implicated in synapse damage in PD, its interaction with mitochondrial complex I, and its role in synaptic function are areas of ongoing research to better understand the mechanisms underlying PD pathogenesis (42). Proteins with a large fold change and a relationship of interest with cell populations (neurons, microglia, and astroglia) or with the pathological α-syn protein and its potential role in PD were validated as DEPs, which included a total of five upregulated DEPs (GNAI1, EEF1A1, PLP1, NPTN, and YWHAH) and two downregulated DEPs (GRIM19 and ORM2) (Figs. 1 and 3).

**Fig. 8:**
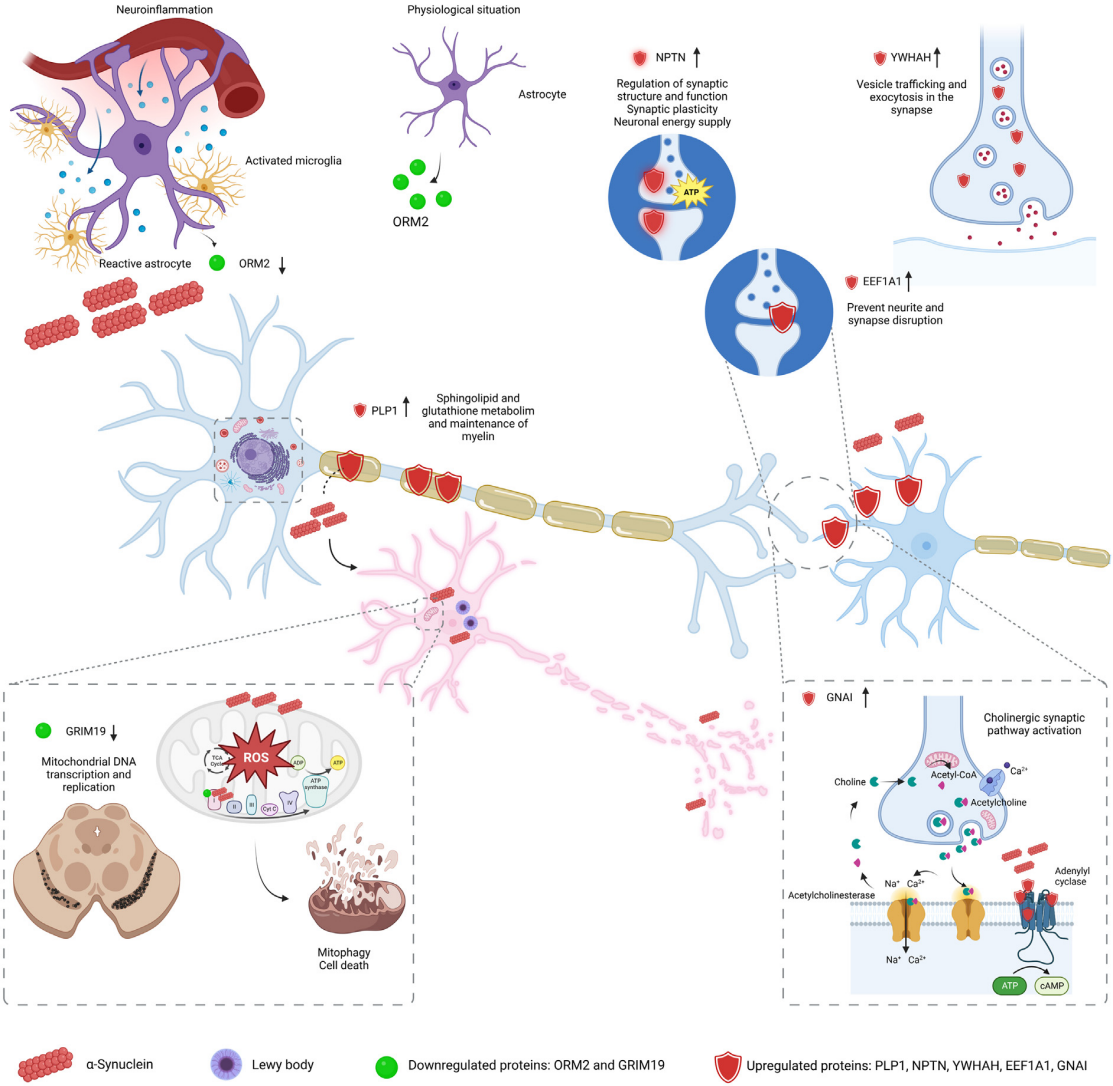
**Overview of the potential role of upregulated GNAI1, EEF1A1, PLP1, NPTN, YWHAH and downregulated GRIM19- and ORM2-validated proteins in Parkinson's disease.** Created using BioRender.com.

The GNAI gene has previously been proposed as a potential target in the diagnosis and treatment of PD and has been reported to be especially upregulated in the striatum and hippocampus of a PD murine model (43). It is a regulator of adenylate cyclase, which plays an important role in the cholinergic synaptic pathway (43) (Fig. 8). Under physiological conditions, the cholinergic pathway promotes the survival of neurons through mitochondrial dysfunction, oxidative stress, and neuroinflammation and controls dopaminergic activity (44). Therefore, this pathway is notably relevant in PD, and its dysfunction contributes not only to cognitive but also to other nonmotor features and motor impairments (45). The striatum, which is a critical node for balancing dopamine signaling and regulating movement, projects cholinergic projections toward the AC (44). Acetylcholine is a two-unit neurotransmitter of this pathway, linking to two kinds of acetylcholine receptors, G-coupled muscarinic acetylcholine receptors, and ionotropic nicotinic receptors (46). In fact, several studies suggest the clinical usefulness of antimuscarinic drugs for treating PD motor symptoms or inhibitors of the enzyme acetylcholinesterase for the treatment of dementia (46). According to previous studies, GNAI protein was also upregulated in the AC of patients with PD (Fig. 3, *A*, *B*, *O* and *P*), which may counteract the cholinergic imbalance.

Although the mechanism by which EEF1A1 contributes to PD is not fully understood, it has been proposed as a gene of interest to understand PD given its important role in the maintenance of synaptic plasticity and in the control of the heat shock response (47) (Fig. 8). Our study revealed that EEF1A1 is upregulated in the AC of patients with PD. Qualitative validation showed such a reduction (Fig. 3, *C* and *D*), even though quantitative Western blot analysis (Fig. 3, *O* and *P*) did not show significant differences between NPD and PD. Our result is consistent with a study in cell cultures demonstrating an increase in EEF1A1 expression after cell exposure to 1-methyl-4-phenylpyridinium (48). According to the data presented, it seems that EEF1A1 could prevent protein, neurite and synapse disruption, and, ultimately, neuronal death (Fig. 8). This agrees with the lack of neurodegeneration reported by stereological results (Fig. 6, *A*–*C*). Indeed, colocalization analyses showed that EEF1A1 labeled neurons immunoreactive for MAP2 (Fig. 4, *A*–*C*). EEF1A1 could be a potential biomarker of PD, as it has also been shown to be upregulated in platelets from PD patients (49).

**Supplemental Figure S2:**
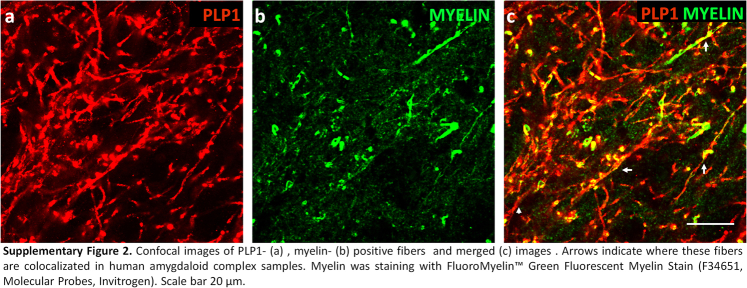


Another protein of diagnostic and therapeutic interest is PLP1 (50). PLP1 participates in sphingolipid and glutathione metabolism and in the maintenance of myelin in the central nervous system (50). Homeostasis of membrane sphingolipids in neurons and myelin is critical to inhibit loss of synaptic plasticity, cell death, and neurodegeneration (51). Under physiological conditions, acid sphingomyelinase and acid ceramidase are enzymes involved in the reduction of α-syn production and aggregation. This metabolism is dysregulated in PD, inducing, among others, the transition of the monomeric form of α-syn to an oligomeric toxic form, even the appearance of LBs (51). In addition, dysfunctional sphingolipids can disrupt the autophagy process, which is responsible for the clearance of misfolded proteins (α-syn) and damaged organelles (52). In our study, PLP1 was upregulated (Fig. 3, *E*, *F*, *O* and *P*) and colocalized with the axonal marker PAN (Fig. 4, *D*–*F*). Most likely, PLP1 is upregulated in PD in order to keep myelin in a nonpathological state that allows rapid long-distance synaptic transmission (Supplemental Fig. S2). In fact, LNs and LBs particularly affect neurons with unmyelinated axons (53), so PLP1 overexpression could be a mechanism to protect against Lewy pathology (Fig. 8). In agreement with our results, a previous study indicated an increase in PLP1 transcripts in the frontal cortex of both mouse and human PD (54). However, in a murine model of PD, the PLP1 protein was downregulated in blood samples (55).

NPTN is part of the presynapse and postsynapse and performs important functions, including the induction of neurite outgrowth and the regulation of synaptic structure and function, synaptic plasticity, and neuronal energy supply (56). Our results indicate NPTN upregulation in the AC of patients with PD (Fig. 3, *G*, *H*, *O* and *P*), which is consistent with previous studies (Fig. 8) (57, 58). Data in rat glial cells have indicated that endoplasmic reticulum stress causes NPTN upregulation, activating the inflammatory response. However, this report also showed that mesencephalic astrocyte-derived neurotrophic factor, an NPTN ligand identified as a protector of dopaminergic neurons, is also upregulated, inhibiting inflammation and preventing cell death (58).

YWHAH, or 14-3-3η, is a member of the 14-3-3 protein family. The 14-3-3 proteins are most abundantly expressed in the brain and are involved in cell signaling, growth, division, adhesion, differentiation, survival, apoptosis, and ion channel regulation (59). Furthermore, the YWHAB, YWHAH, and YWHAE isoforms have been reported to interact with α-syn, and colocalization between 14-3-3 proteins and LBs has been described (60). However, the YWHAH isoform has not been detected in LBs by immunohistochemical assay (61). A study in cell cultures of dopaminergic neurons showed that inducing oxidative stress downregulated the YWHAH gene (59). Our results indicate an increase in YWHAH protein in the AC of patients with PD, which was validated at a qualitative level (Fig. 3, *I* and *J*); however, at a quantitative level, it did not show a significant increase in PD cases (Fig. 3, *O* and *P*). In addition, colocalization analysis showed its position in the nucleus of MAP2-positive neurons (Fig. 4, *G*–*I*). We hypothesized that during PD, YWHAH is upregulated so that its chaperone activity maintains cell survival and controls cell death. YWHAH may even mediate vesicle trafficking and exocytosis at the synaptic level (61) (Fig. 8).

Mitochondrial dysfunction is an inherent mechanism of PD, contributing in different ways: leading to impaired energy metabolism and consequently to an increase in reactive oxygen species, impairing mitochondrial quality control, and impairing calcium homeostasis, contributing to the loss of dopaminergic neurons in PD (62). Deficiency of GRIM19, a subunit of respiratory electron chain complex 1, has been associated with lower rates of mitochondrial DNA transcription and replication in the *substantia nigra* in PD (63). The present study confirms the downregulation of GRIM19 in the affected AC during PD. The qualitative validation by immunofluorescence showed a clear reduction (Fig. 3, *K* and *L*) that was not significant in the quantitative validation by Western blot (Fig. 3, *O* and *P*). Therefore, GRIM19 deficiency in the AC of patients with PD could contribute to mitochondrial dysfunction and consequent oxidative stress (Fig. 8). According to this result, α-syn also interacts with the subunit of respiratory electron chain complex 1 and induces its inhibition (42, 62), playing an important role in the pathogenesis of PD.

Another characteristic process of PD is microglia-mediated neuroinflammation, which is a complex network of interactions comprising immune and nonimmune cells, adjunct to mediators of the immune response, contributing to the progression of this disease (64). Among these immunomodulators, ORM2, which is preferentially expressed in the brain and predominantly synthesized by astrocytes, plays an important role (65). However, our study did not show an exacerbated colocalization between ORM2 and GFAP (Fig. 4, *J*–*L*), which could be explained by ORM2 secretion. In addition, the present study showed that ORM2 is downregulated in the AC of patients with PD (Fig. 3, *M*–*P*). It is possible that astrocytes decrease ORM2 production during PD to stimulate the activity of microglia already exhausted from coping with LBs and LNs (Fig. 8).

Therefore, α-syn can induce toxicity (cell death) in different contexts, namely the reduction of the GRIM19 protein together with blocking complex I in the mitochondria by α-syn contributes to increase reactive oxygen species production and decrease ATP synthesis. In addition, neuroinflammation may potentiate α-syn dysfunction, driving chronic progression of neurodegeneration by inducing astrocytes and microglia reaction, enhanced by ORM2 reduction. However, seeding and spreading, *via* endocytosis to neighboring neurons, could be slowed down by NPTN, EEF1A1, and YWHAH, which could preserve the homeostasis of the neuron, preventing the neurites disruption and aberrant vesicle trafficking. Other protective mechanisms could be the preservation of myelin (PLP1) and the cholinergic synapse (GNAI) (Fig. 8).

On the other hand, these proteins have a key characteristic that makes them interesting for further research as biomarkers. They have been detected in human body fluids such as cerebrospinal fluid, in the case of GNAI1, EEF1A1, PLP1, NPTN, YWHAH, and ORM2, or urine, in the case of GRIM19, which would facilitate their use as potential biomarkers of interest in PD.

The results of the proteomic analysis indicate that the pathology of AC with PD causes proteomic alterations at the synaptic level. This synaptic damage could go further and lead to alterations in the cell populations that comprise them. To determine the effect that the pathology has on the neuronal and glial populations and on the volume of the AC and its nuclear groups, a stereological study was carried out.

According to the literature, LN and LB inclusions are differentially distributed among amygdaloid nuclei (25, 28). A semiquantitative analysis showed that the Co and Ce nuclei were affected earlier and more severely than the basal nucleus (25). The prion-like hypothesis allows us to explain these nuclear differences (66). However, our results indicate that the fraction of area occupied by α-syn does not differ between AC nuclei (Fig. 5), which is in consonance with a previous study by our group (26). The advanced stages of the cases used could explain this pathological homogeneity, as the entire AC is seriously affected in advanced stages of PD. Additionally, there is a positive correlation between the duration of PD and the area fraction occupied by α-syn (Supplemental Fig. S1).

Another semiquantitative study indicated that the percentage of neurons (marked with Nissl staining) with LBs is higher in the cortical and basolateral nuclei (28) and related with the death of the neuronal population (28). Nevertheless, our results revealed that the neuronal population of the AC and its nuclei was not altered in PD (Fig. 6, *A*–*C*). In addition to stereological techniques, the use of neuron-specific labeling minimizes counting errors compared to Nissl staining (67). As previously suggested, some cells can survive LN and LB impairment for a long time but probably cease functioning long before dying (53).

The presence of astrocytes with α-syn aggregates in the AC of patients with PD has been described (14, 15, 41) as well as the activation of microglia in PD. However, this is the first study to analyze microglial (Fig. 6, *D*–*F*) and astroglial (Fig. 6, *G*–*I*) density in the AC of patients with PD, reveling that remain stable in the AC and its nuclei. Whether microglia and astroglia favor the spread of Lewy pathology or, in contrast, eliminate or retain LNs and LBs, avoiding neuronal involvement is still unknown (68, 69, 70, 71).

Potential changes in cell populations (gray matter) and/or their connections (white matter) can alter the volume measurable by imaging techniques, which can be used to analyze the total volume of the AC, but stereological approaches are more costly and allow the delimitation of AC nuclear complexes. A single stereological study analyzed the AC volume of patients with PD and found AC atrophy due to volume reduction in the corticomedial complex, in which higher neuronal loss has been reported (28). Our results indicated that the volumes of the AC and its nuclei did not vary between PD and NPD cases (Fig. 7). We consider that although the damage caused by LNs and LBs is not sufficient to lead to the loss of gray and/or white matter, it could damage cell functionality, as revealed by proteomic data.

## Conclusions

To our knowledge, this is the first proteomic study of the AC of patients with PD, and it identified possible biomarkers of the disease. Considering the data reported here, it seems clear that Lewy pathology is not sufficient to cause neurodegeneration or alteration of microglial and astroglial populations in the AC of humans with PD. However, damage at the proteomic level is evident, highlighting significant synaptic involvement.

## Ethics approval and consent to participate

All experiments were performed in accordance with the Clinical Research Ethics Committee of the University Hospital of Ciudad Real (PID2019-108659RB-I00).

## Data Availability

The mass spectrometry proteomics data are available in the ProteomeXchange Consortium (http://proteomecentral.proteomexchange.org) *via* the PRIDE partner repository with the dataset identifier PXD043503 (Username: reviewer_pxd043503@ebi.ac.uk; Password: eHWcHRb6).

Other data generated or analyzed during this study are included in this published article and its supplementary information files are available from the corresponding author upon reasonable request.

DIANN does not generate annotated spectrum files but.tsv files, which are results files. Alphamap (https://github.com/MannLabs/alphamap) allows you to visualize the sequences and peptides identified by DIANN for each identified protein. AlphaViz (https://github.com/MannLabs/alphaviz) is an automated visualization pipeline to link these identifications with the original raw data and easily assess their individual quality or the overall quality whole samples.

## Supplemental data

This article contains supplemental data.

## Conflict of interest

The authors declare that they have no competing interests.

## Supplementary Data

Supplemental Data S1 *A*, List of the identified proteins in the spectral library. Each protein is identified by its ID code, gene name, number of unique peptides and protein sequence coverage. *B*, Dia-PASEF identified proteins list. The protein ID code, gene name, full protein name, number of unique peptides, protein sequence coverage and protein quantification information are displayed for each sample. *C*, Quantified amygdaloid complex proteome in the dia-PASEF analysis. For each sample, the protein ID code, gene name, full protein name, number of unique peptides, protein sequence coverage and protein counting information are shown. *D*, Heatmap produced from a list of quantified proteins with a *p*-value cutoff ≤ 0.05. The proteins' ID codes, gene names, full names, the number of unique peptides, the p-value for the t-test comparing the Parkinson’s disease and non-Parkinson’s disease groups, and the quantification data for each protein are all given. *E*, List of differentially expressed proteins filtered by fold change (≥ 1.3 and ≤ 0.77) and *p*-value (≤ 0.05). There are indications for each protein: ID code, gene name, full name protein, number unique peptides, *p*-value, fold change represented in a red/green column of increasing color and quantification data. *F*, List of the 25 α-synuclein-interacting proteins that are differentially expressed in the amygdala complex in Parkinson's disease. The list was created by comparing (in Venny 2.1, https://bioinfogp.cnb.csic.es/tools/venny/) the 562 proteins that interact with α-synuclein (from BioGRID 4.4.211, https://thebiogrid.org/) and the 199 differentially expressed proteins from proteomic analysis. *G*, List of the 46 differentially expressed proteins that have synaptic gene ontology annotations (SynGO 20210225, www.syngoportal.org). For each protein, the GO term name and GO domain CC, cellular component, BP, biological process are provided. *H*, IPA analysis reveals disease and function annotations that are altered (activated or decreased) as a result of upregulation or downregulation of differentially expressed proteins (DEPs) in the human amygdaloid complex (AC) in Parkinson's disease (PD).
