# Supplementary material for: Synaptic Involvement of the Human Amygdala in Parkinson’s Disease
Source: Mol Cell Proteomics. 2023 Oct 29;22(12):100673. doi: 10.1016/j.mcpro.2023.100673 (PMC10700869; doi:10.1016/j.mcpro.2023.100673)

**Supplementary Data 2.** Supplementary legend for Figure 4. If necessary, a complete legend is available in https://qiagen.my.salesforce-sites.com/KnowledgeBase/articles/Knowledge/Legend/p

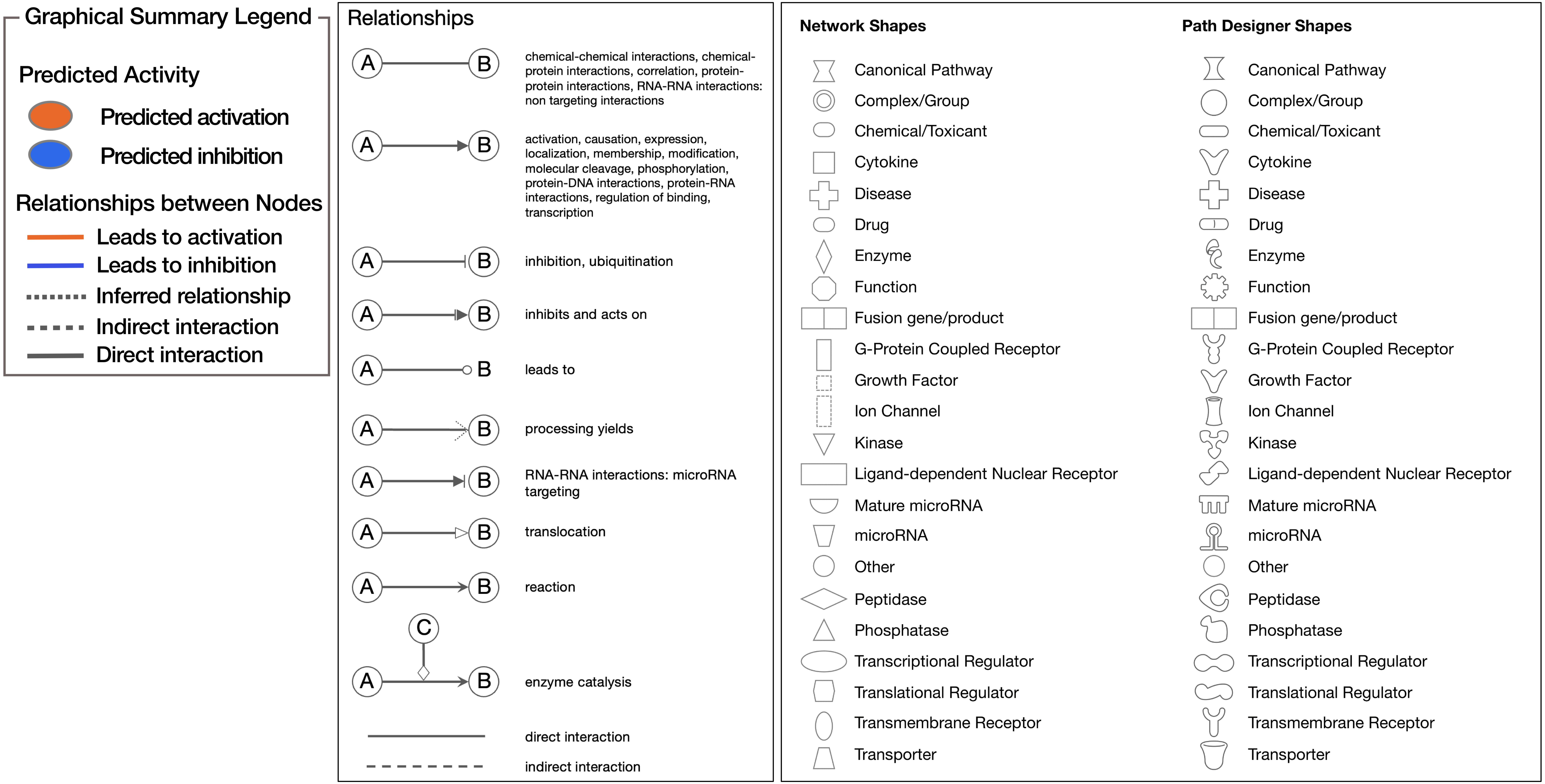

Supplement: Supplemental Data S2 [file mmc2.pdf]
