# Supplementary material for: Synaptic Involvement of the Human Amygdala in Parkinson’s Disease
Source: Mol Cell Proteomics. 2023 Oct 29;22(12):100673. doi: 10.1016/j.mcpro.2023.100673 (PMC10700869; doi:10.1016/j.mcpro.2023.100673)
Supplement: Supplemental Tables S1–S5 [file mmc3.pdf]

Synaptic involvement of the human amygdala in Parkinson's disease

Sandra Villar-Conde[1,2], Veronica Astillero-Lopez[1,2], Melania Gonzalez-Rodriguez[1,2], Daniel Saiz-Sanchez[1,2], Alino Martinez-Marcos[1,2]*, Isabel Ubeda-Banon[1,2]*, Alicia Flores-Cuadrado[1,2].

[1]Grupo de Neuroplasticidad y Neurodegeneración, CRIB, Facultad de Medicina de Ciudad Real, Universidad de Castilla-La Mancha (UCLM), Spain.
[2]Grupo de Neuroplasticidad y Neurodegeneración, Instituto de Investigación Sanitaria de Castilla-La Mancha (IDISCAM), Spain.

Supplementary table 1. Area fraction occupied by α-syn.

Supplementary table 2: Estimated MAP2-positive cells number and density.

Supplementary table 3: Estimated Iba-1-positive cells number and density.

Supplementary table 4: Estimated GFAP-positive cells number and density.

Supplementary table 5. Volume data.

Supplementary table 1. Area fraction occupied by α-syn.

Supplementary table 1a. Area fraction occupied by α-syn in the basolateral nuclear group.

Case	Number of Sections	Section Cut Thickness (µm)	Section Evaluation Interval	Counting Frame Area (XY) (µm²)	Sampling Grid Area (XY) (µm²)	Grid Spacing (µm)	Number of Sampling Sites	Total Markers Counted	Area Sampling Fraction	Area Fraction	Estimated area (µm²)	Coefficient of Error (Gundersen), m=1
17	4	50	13	10,000	2,250,000	10	186	11	0.0044	0.0006	247,500	0.14
18	4	50	13	10,000	2,250,000	10	193	8	0.0044	0.0004	180,000	0.19
19	4	50	13	10,000	2,250,000	10	162	86	0.0044	0.0054	1,935,000	0.03
20	4	50	13	10,000	2,250,000	10	170	53	0.0044	0.0032	1,192,500	0.07
21	4	50	13	10,000	2,250,000	10	172	41	0.0044	0.0025	922,500	0.06
22	4	50	13	10,000	2,250,000	10	147	18	0.0044	0.0013	405,000	0.11
23	4	50	13	10,000	2,250,000	10	147	6	0.0044	0.0004	135,000	0.19
24	4	50	13	10,000	2,250,000	10	113	31	0.0044	0.0029	697,500	0.07
25	4	50	13	10,000	2,250,000	10	102	26	0.0044	0.0026	585,000	0.09
26	4	50	13	10,000	2,250,000	10	140	16	0.0044	0.0012	360,000	0.10

Supplementary table 1b. Area fraction occupied by α-syn in the cortical nuclear group.

Case	Number of Sections	Section Cut Thickness (µm)	Section Evaluation Interval	Counting Frame Area (XY) (µm²)	Sampling Grid Area (XY) (µm²)	Grid Spacing (µm)	Number of Sampling Sites	Total Markers Counted	Area Sampling Fraction	Area Fraction	Estimated area (µm²)	Coefficient of Error (Gundersen), m=1
17	4	50	13	10,000	360,000	10	190	20	0.0278	0.0011	72,000	0.08
18	4	50	13	10,000	360,000	10	185	8	0.0278	0.0005	28,800	0.17
19	4	50	13	10,000	360,000	10	122	74	0.0278	0.0064	266,400	0.05
20	4	50	13	10,000	360,000	10	159	46	0.0278	0.0031	165,600	0.06
21	4	50	13	10,000	360,000	10	103	23	0.0278	0.0024	82,800	0.09
22	4	50	13	10,000	360,000	10	156	31	0.0278	0.0021	111,600	0.10
23	4	50	13	10,000	360,000	10	171	1	0.0278	0.0001	3,600	0.78
24	4	50	13	10,000	360,000	10	141	40	0.0278	0.0016	144,000	0.07
25	4	50	13	10,000	360,000	10	118	17	0.0278	0.0016	61,200	0.10
26	3	50	13	10,000	360,000	10	77	12	0.0278	0.0017	43,200	0.13

Supplementary table 1c. Area fraction occupied by α-syn in the central nuclear group.

Case	Number of Sections	Section Cut Thickness (µm)	Section Evaluation Interval	Counting Frame Area (XY) (µm²)	Sampling Grid Area (XY) (µm²)	Grid Spacing (µm)	Number of Sampling Sites	Total Markers Counted	Area Sampling Fraction	Area Fraction	Estimated area (µm²)	Coefficient of Error (Gundersen), m=1
17	4	50	13	10,000	160,000	10	298	11	0.0625	0.0004	17,600	0.14
18	4	50	13	10,000	160,000	10	266	4	0.0625	0.0002	6,400	0.27
19	4	50	13	10,000	160,000	10	187	70	0.0625	0.0042	112,000	0.04
20	4	50	13	10,000	160,000	10	203	20	0.0625	0.0011	32,000	0.08
22	4	50	13	10,000	160,000	10	237	16	0.0625	0.0007	25,600	0.10
23	2	50	13	10,000	160,000	10	86	3	0.0625	0.0004	4,800	0.28
26	4	50	13	10,000	160,000	10	198	18	0.0625	0.0010	28,800	0.09

Supplementary table 2: Estimated MAP2-positive cells number and density.

Supplementary table 2a: Estimated neuronal number and density of the basolateral nuclear group.

Case	Number of Sections	Section Cut Thickness (µm)	Section Evaluation Interval	Disector Height (Z) (µm)	Guard Zone Distance (µm)	Mean Measured Section Thickness (µm)	Counting Frame Area (XY) (µm^2)	Sampling Grid Area (XY) (µm^2)	Number of Sampling Sites	Total Markers Counted	Estimated Population using Mean Section Thickness	Measured Volume (mm^3)	Coefficient of Error (Gundersen). m=1	Density (cell/mm^3)
17	4	50	13	9	2	13	2,500	1,690,000	219	150	1,905,910.25	235	0.09	8,126.75
18	4	50	13	9	2	13	2,500	2,890,000	150	114	2,477,977.25	264	0.10	9,403.05
19	4	50	13	9	2	13	2,500	1,690,000	220	160	2,029,426.13	239	0.08	8,483.16
20	4	50	13	9	2	13	2,500	1,440,000	243	145	1,568,871.63	224	0.09	6,991.03
21	4	50	13	9	2	13	2,500	2,890,000	134	123	2,689,635.75	247	0.10	10,874.29
22	4	50	13	9	2	13	2,500	1,690,000	232	125	1,573,592.88	246	0.09	6,388.46
23	4	50	13	9	2	13	2,500	1,960,000	166	138	2,029,968.88	204	0.09	9,949.75
24	4	50	13	9	2	13	2,500	1,960,000	150	123	1,809,788.75	188	0.09	9,602.48
25	4	50	13	9	2	13	2,500	1,960,000	117	119	1,749,271.63	145	0.09	12,045.00
26	4	50	13	9	2	13	2,500	1,690,000	191	149	1,892,188.38	208	0.09	9,086.58
27	4	50	13	9	2	13	2,500	1,690,000	257	179	3,321,522.75	400	0.08	8,299.06
28	4	50	13	9	2	13	2,500	1,690,000	170	127	1,611,507.63	181	0.09	8,908.92
29	4	50	13	9	2	13	2,500	1,440,000	236	140	1,516,430.25	212	0.09	7,156.82
30	4	50	13	9	2	13	2,500	1,210,000	319	151	1,373,772.13	246	0.09	5,587.98
31	4	50	13	9	2	13	2,500	2,560,000	199	126	2,424,843.00	326	0.09	7,429.80
32	4	50	13	9	2	13	2,500	1,960,000	195	160	2,355,639.50	237	0.08	9,938.53
33	4	50	13	9	2	13	2,500	1,690,000	180	125	1,586,941.75	188	0.09	8,440.01
34	4	50	13	9	2	13	2,500	810,000	155	112	681,902.56	80	0.10	8,530.66
35	4	50	13	9	2	13	2,500	1,440,000	166	114	1,234,024.13	152	0.10	8,099.02
36	4	50	13	9	2	13	2,500	1,690,000	144	107	1,359,364.88	157	0.10	8,643.90

Supplementary table 2b: Estimated neuronal number and density of the cortical nuclear group.

Case	Number of Sections	Section Cut Thickness (µm)	Section Evaluation Interval	Disector Height (Z) (µm)	Guard Zone Distance (µm)	Mean Measured Section Thickness (µm)	Counting Frame Area (XY) (µm^2)	Sampling Grid Area (XY) (µm^2)	Number of Sampling Sites	Total Markers Counted	Estimated Population using Mean Section Thickness	Measured Volume (mm^3)	Coefficient of Error (Gundersen). m=1	Density (cell/mm^3)
17	4	50	13	9	2	13	2,500	202,500	169	132	200,847.33	22	0.09	9,312.33
18	4	50	13	9	2	13	2,500	360,000	139	136	368,260.69	31	0.09	11,920.29
19	4	50	13	9	2	13	2,500	360,000	132	137	370,350.13	30	0.09	12,246.50
20	4	50	13	9	2	13	2,500	250,000	231	136	255,792.84	37	0.09	6,960.37
21	4	50	13	9	2	13	2,500	250,000	125	130	243,442.16	19	0.09	12,796.24
22	4	50	13	9	2	13	2,500	250,000	192	111	208,339.23	30	0.10	7,024.46
23	4	50	13	9	2	13	2,500	250,000	170	147	276,247.75	26	0.09	10,768.30
24	4	50	13	9	2	13	2,500	360,000	124	99	267,626.00	27	0.10	10,086.15
25	4	50	13	9	2	13	2,500	302,500	131	164	372,227.16	24	0.08	15,393.18
27	4	50	13	9	2	13	2,500	202,500	242	169	257,386.69	31	0.08	8,247.19
28	4	50	13	9	2	13	2,500	202,500	197	142	215,891.28	25	0.09	8,603.51
29	4	50	13	9	2	13	2,500	160,000	309	190	228,614.42	32	0.08	7,212.27
30	4	50	13	9	2	13	2,500	160,000	289	135	162,249.86	29	0.09	5,677.58
31	4	50	13	9	2	13	2,500	160,000	230	141	169,400.19	23	0.09	7,343.86
32	4	50	13	9	2	13	2,500	160,000	192	197	237,071.09	19	0.08	12,246.99
33	4	50	13	9	2	13	2,500	250,000	185	147	276,128.59	29	0.09	9,444.65
34	4	50	13	9	2	13	2,500	160,000	227	178	212,488.55	23	0.08	9,383.22
35	3	50	13	9	2	13	2,500	160,000	197	152	182,975.19	19	0.09	9,555.54
36	4	50	13	9	2	13	2,500	202,500	160	137	208,873.28	19	0.09	10,837.61

Supplementary table 2c: Estimated neuronal number and density of the central nuclear group.

Case	Number of Sections	Section Cut Thickness (µm)	Section Evaluation Interval	Disector Height (Z) (µm)	Guard Zone Distance (µm)	Mean Measured Section Thickness (µm)	Counting Frame Area (XY) (µm²)	Sampling Grid Area (XY) (µm²)	Number of Sampling Sites	Total Markers Counted	Estimated Population using Mean Section Thickness	Measured Volume (mm³)	Coefficient of Error (Gundersen). m=1	Density (cell/mm³)
17	4	50	13	9	2	13	2,500	90,000	222	141	95,372.74	12	0.09	8,137.26
18	4	50	13	9	2	13	2,500	90,000	282	127	86,003.23	15	0.09	5,726.83
19	4	50	13	9	2	13	2,500	90,000	266	156	105,486.35	15	0.09	7,084.19
20	4	50	13	9	2	13	2,500	90,000	198	107	72,399.83	11	0.10	6,725.73
21	4	50	13	9	2	13	2,500	40,000	126	113	33,992.67	03	0.10	11,535.45
22	4	50	13	9	2	13	2,500	62,500	335	135	63,008.54	13	0.10	4,998.85
23	4	50	13	9	2	13	2,500	90,000	229	194	131,271.69	12	0.07	10,695.89
24	3	50	13	9	2	13	2,500	62,500	170	126	59,244.78	07	0.09	8,837.48
26	4	50	13	9	2	13	2,500	90,000	123	135	91,324.67	07	0.10	13,855.86
27	4	50	13	9	2	13	2,500	160,000	206	112	134,738.31	20	0.10	6,595.25
29	4	50	13	9	2	13	2,500	10,000	977	198	14,894.82	06	0.08	2,633.72
30	2	50	13	9	2	13	2,500	15,625	371	163	19,154.69	03	0.09	5,528.61
31	4	50	13	9	2	13	2,500	62,500	296	139	65,345.17	11	0.09	5,788.03
32	4	50	13	9	2	13	2,500	160,000	157	110	132,178.31	15	0.10	8,634.08
33	4	50	13	9	2	13	2,500	22,500	237	157	26,587.44	03	0.09	8,822.81
34	4	50	13	9	2	13	2,500	40,000	325	186	55,825.37	08	0.09	7,327.64
35	4	50	13	9	2	13	2,500	122,500	185	133	122,266.81	13	0.09	9,187.12
36	4	50	13	9	2	13	2,500	122,500	161	153	140,899.69	12	0.08	11,493.57

Supplementary table 3: Estimated Iba-1-positive cells number and density.

Supplementary table 3a: Estimated microglia number and density of the basolateral nuclear group.

Case	Number of Sections	Section Cut Thickness (µm)	Section Evaluation Interval	Disector Height (Z) (µm)	Guard Zone Distance (µm)	Mean Measured Section Thickness (µm)	Counting Frame Area (XY) (µm²)	Sampling Grid Area (XY) (µm²)	Number of Sampling Sites	Total Markers Counted	Estimated Population using Mean Section Thickness	Measured Volume (mm³)	Coefficient of Error (Gundersen). m=1	Density (cell/mm³)
17	4	50	13	9	2	13	2,500	3,240,000	116	106	2,581,600.75	233	0.10	11,071.33
18	2	50	13	9	2	13	2,500	1,000,000	190	151	1,113,015.00	123	0.09	9,070.51
19	4	50	13	9	2	13	2,500	2,250,000	152	155	2,624,375.50	220	0.08	11,914.79
20	4	50	13	9	2	13	2,500	1,000,000	360	112	842,079.75	235	0.10	3,578.25
21	4	50	13	9	2	13	2,500	2,250,000	150	182	3,075,289.25	218	0.08	14,084.48
22	4	50	13	9	2	13	2,500	2,250,000	137	105	1,775,352.75	190	0.10	9,322.28
23	4	50	13	9	2	13	2,500	1,000,000	219	111	834,342.00	139	0.90	5,982.49
24	4	50	13	9	2	13	2,500	1,690,000	135	130	1,648,513.38	137	0.09	12,029.26
25	4	50	13	9	2	13	2,500	1,690,000	126	130	1,653,177.25	131	0.07	12,646.12
26	4	50	13	9	2	13	2,500	1,960,000	146	104	1,536,279.38	181	0.10	8,469.81
27	4	50	13	9	2	13	2,500	1,210,000	315	110	999,364.75	247	0.10	4,050.36
28	4	50	13	9	2	13	2,500	1,960,000	153	124	1,824,792.13	193	0.09	9,465.92
29	4	50	13	9	2	13	2,500	1,960,000	163	117	1,722,613.13	202	0.09	8,530.24
30	4	50	13	9	2	13	2,500	490,000	688	118	434,249.91	218	0.10	1,992.74
31	4	50	13	9	2	13	2,500	3.240.000	135	131	3190767.50	277	0.09	11518.64
32	4	50	13	9	2	13	2,500	1.000.000	316	115	861885.19	203	0.10	4243.36
33	4	50	13	9	2	13	2,500	2.250.000	137	123	2077273.88	197	0.09	10552.09
34	4	50	13	9	2	13	2,500	1.000.000	138	151	1133130.50	87	0.09	13020.57
35	4	50	13	9	2	13	2,500	1.440.000	149	178	1913491.13	142	0.08	13432.16
36	4	50	13	9	2	13	2,500	1.690.000	172	139	1760991.63	183	0.09	9602.96

Supplementary table 3b: Estimated microglia number and density of the cortical nuclear group.

Case	Number of Sections	Section Cut Thickness (µm)	Section Evaluation Interval	Disector Height (Z) (µm)	Guard Zone Distance (µm)	Mean Measured Section Thickness (µm)	Counting Frame Area (XY) (µm²)	Sampling Grid Area (XY) (µm²)	Number of Sampling Sites	Total Markers Counted	Estimated Population using Mean Section Thickness	Measured Volume (mm³)	Coefficient of Error (Gundersen). m=1	Density (cell/mm³)
17	4	50	13	9	2	13	2,500	490,000	110	119	438,343.81	33	0.09	13,419.04
18	2	50	13	9	2	13	2,500	122,500	195	189	170,656.17	15	0.09	11,482.10
19	4	50	13	9	2	13	2,500	360,000	150	202	545,926.75	34	0.08	16,268.69
20	4	50	13	9	2	13	2,500	302,500	157	124	281,663.50	30	0.09	9,438.27
21	4	50	13	9	2	13	2,500	302,500	132	198	434,955.81	25	0.08	17,585.27
22	4	50	13	9	2	13	2,500	360,000	148	121	327,501.72	34	0.10	9,723.49
23	4	50	13	9	2	13	2,500	360,000	143	157	424,707.75	33	0.08	13,066.48
24	4	50	13	9	2	13	2,500	360,000	132	145	391,885.09	29	0.09	13,541.06
25	4	50	13	9	2	13	2,500	250,000	146	164	307,927.09	22	0.08	13,754.67
27	4	50	13	9	2	13	2,500	640,000	106	144	691,691.31	44	0.09	15,638.65
28	4	50	13	9	2	13	2,500	302,500	157	124	281,663.50	30	0.09	9,438.27
29	4	50	13	9	2	13	2,500	360,000	149	124	335,500.78	33	0.09	10,060.08
30	4	50	13	9	2	13	2,500	360,000	163	130	351,606.94	38	0.09	9,375.19
31	4	50	13	9	2	13	2,500	490,000	144	148	545,062.75	42	0.08	12,827.93
32	2	50	13	9	2	13	2,500	160,000	165	127	151,719.08	17	0.10	9,185.13
33	3	50	13	9	2	13	2,500	160,000	144	187	225,011.22	14	0.08	15,808.23
34	4	50	13	9	2	13	2,500	250,000	146	243	455,970.63	23	0.07	20,025.41
35	4	50	13	9	2	13	2,500	250,000	152	221	411,112.53	23	0.07	17,735.66
36	4	50	13	9	2	13	2,500	250,000	163	158	296,383.28	25	0.08	11,727.32

Supplementary table 3c: Estimated microglia number and density of the central nuclear group.

Case	Number of Sections	Section Cut Thickness (µm)	Section Evaluation Interval	Disector Height (Z) (µm)	Guard Zone Distance (µm)	Mean Measured Section Thickness (µm)	Counting Frame Area (XY) (µm²)	Sampling Grid Area (XY) (µm²)	Number of Sampling Sites	Total Markers Counted	Estimated Population using Mean Section Thickness	Measured Volume (mm³)	Coefficient of Error (Gundersen). m=1	Density (cell/mm³)
17	4	50	13	9	2	13	2,500	160,000	210	157	188,988.78	21	0.09	9,158.61
18	2	50	13	9	2	13	2,500	90,000	189	130	86,240.24	10	0.10	8,221.58
19	4	50	13	9	2	13	2,500	160,000	140	138	166,195.66	14	0.09	11,765.40
20	4	50	13	9	2	13	2,500	62,500	381	96	45,081.47	15	0.10	3,058.73
22	4	50	13	9	2	13	2,500	122,500	247	127	116,850.78	19	0.09	6,289.20
23	4	50	13	9	2	13	2,500	90,000	373	113	76,385.50	21	0.10	3,687.95
26	4	50	13	9	2	13	2,500	160,000	198	107	128,931.32	20	0.10	6,428.37
27	4	50	13	9	2	13	2,500	90,000	546	131	88,486.11	31	0.09	2,872.92
28	4	50	13	9	2	13	2,500	202,500	155	118	177,691.22	20	0.09	8,762.54
29	4	50	13	9	2	13	2,500	250,000	118	99	186,171.98	18	0.10	10,212.68
31	4	50	13	9	2	13	2,500	250,000	119	105	197,307.55	18	0.10	10,671.04
32	4	50	13	9	2	13	2,500	90,000	370	148	99,876.95	21	0.09	4,851.62
35	4	50	13	9	2	13	2,500	360,000	119	117	314,542.88	26	0.10	11,958.99
36	4	50	13	9	2	13	2,500	302,500	165	112	254,080.00	30	0.10	8,391.63

Supplementary table 4: Estimated GFAP-positive cells number and density.

Supplementary table 4a: Estimated astroglial number and density of the basolateral nuclear group.

Case	Number of Sections	Section Cut Thickness (µm)	Section Evaluation Interval	Disector Height (Z) (µm)	Guard Zone Distance (µm)	Mean Measured Section Thickness (µm)	Counting Frame Area (XY) (µm²)	Sampling Grid Area (XY) (µm²)	Number of Sampling Sites	Total Markers Counted	Estimated Population using Mean Section Thickness	Measured Volume (mm³)	Coefficient of Error (Gundersen). m=1	Density (cell/mm³)
17	4	50	13	9	2	13	2,500	1,000,000	365	134	1,005,550.50	228	0.09	4,406.27
18	4	50	13	9	2	13	2,500	1,000,000	362	117	877,834.38	233	0.10	3,774.41
19	4	50	13	9	2	13	2,500	2,250,000	146	107	1,807,863.13	214	0.10	8,429.72
20	4	50	13	9	2	13	2,500	1,000,000	375	121	907,379.25	243	0.09	3,731.94
21	4	50	13	9	2	13	2,500	2,250,000	149	129	2,180,019.25	208	0.09	10,461.50
22	3	50	13	9	2	13	2,500	1,000,000	226	104	781,188.75	145	0.10	5,391.00
23	4	50	13	9	2	13	2,500	640,000	466	147	707,181.75	185	0.08	3,817.94
24	4	50	13	9	2	13	2,500	1,440,000	178	108	1,164,449.75	163	0.10	7,152.51
25	4	50	13	9	2	13	2,500	1,210,000	197	102	926,437.81	150	0.10	6,174.19
26	4	50	13	9	2	13	2,500	1,000,000	299	95	711,862.94	188	0.10	3,785.58
27	4	50	13	9	2	13	2,500	1,210,000	302	178	1614032.00	236	0.08	6,843.18
28	4	50	13	9	2	13	2,500	1,000,000	282	115	862,409.25	177	0.10	4,879.51
29	4	50	13	9	2	13	2,500	1,000,000	307	97	727,926.00	198	0.10	3,677.34
30	4	50	13	9	2	13	2,500	1,440,000	261	119	1,287,252.13	240	0.09	5,366.44
31	4	50	13	9	2	13	2,500	40,000	779	112	33,628.50	19	0.10	1,732.63
32	4	50	13	9	2	13	2,500	160,000	2154	112	134,022.33	219	0.10	,613.22
33	4	50	13	9	2	13	2,500	1,960,000	161	96	1,412,057.75	200	0.10	7,066.12
34	4	50	13	9	2	13	2,500	490,000	240	145	532,775.06	76	0.09	7,042.45
35	4	50	13	9	2	13	2,500	360,000	641	177	473,228.25	147	0.08	3,228.68
36	4	50	13	9	2	13	2,500	640,000	465	174	836,355.94	191	0.08	4,374.32

Supplementary table 4b: Estimated astroglial number and density of the cortical nuclear group.

Case	Number of Sections	Section Cut Thickness (µm)	Section Evaluation Interval	Disector Height (Z) (µm)	Guard Zone Distance (µm)	Mean Measured Section Thickness (µm)	Counting Frame Area (XY) (µm²)	Sampling Grid Area (XY) (µm²)	Number of Sampling Sites	Total Markers Counted	Estimated Population using Mean Section Thickness	Measured Volume (mm³)	Coefficient of Error (Gundersen). m=1	Density (cell/mm³)
17	4	50	13	9	2	13	2,500	250,000	227	98	183,857.86	35	0.09	5,185.23
18	4	50	13	9	2	13	2,500	250,000	255	102	191,260.56	40	0.10	4,835.33
19	4	50	13	9	2	13	2,500	250,000	197	116	217,652.14	31	0.10	7,134.52
20	4	50	13	9	2	13	2,500	90,000	614	114	77,007.89	35	0.10	2,228.33
21	4	50	13	9	2	13	2,500	250,000	158	107	200,975.17	25	0.10	8,175.11
22	3	50	13	9	2	13	2,500	122,500	332	127	115,152.84	25	0.09	4,536.06
23	4	50	13	9	2	13	2,500	40,000	1181	195	58,506.64	30	0.09	1,983.26
24	4	50	13	9	2	13	2,500	160,000	299	118	141,657.03	31	0.09	4,627.24
25	4	50	13	9	2	13	2,500	90,000	514	157	105,973.37	28	0.08	3,792.96
26	3	50	13	9	2	13	2,500	5,625	2595	214	9,027.45	09	0.08	1,048.26
27	4	50	13	9	2	13	2,500	202,500	297	133	202,014.69	38	0.09	5,349.68
28	3	50	13	9	2	13	2,500	160,000	338	163	195,643.28	34	0.08	5,802.39
29	4	50	13	9	2	13	2,500	202,500	283	108	164,138.67	36	0.10	4,550.98
30	4	50	13	9	2	13	2,500	160,000	452	157	188,670.00	46	0.08	4,142.55
31	4	50	13	9	2	13	2,500	62,500	958	103	48,336.21	38	0.10	1,285.44
32	3	50	13	9	2	13	2,500	40,000	105	168	50,058.23	25	0.09	2,004.81
33	4	50	13	9	2	13	2,500	90,000	288	117	78,991.27	16	0.10	4,900.84
34	4	50	13	9	2	13	2,500	202,500	174	128	194,516.69	21	0.09	9,124.40
35	4	50	13	9	2	13	2,500	90,000	449	128	85,862.72	25	0.10	3,472.51
36	4	50	13	9	2	13	2,500	90,000	384	116	78,420.53	22	0.09	3,641.07

Supplementary table 4c: Estimated astroglial number and density of the central nuclear group.

Case	Number of Sections	Section Cut Thickness (μm)	Section Evaluation Interval	Disector Height (Z) (μm)	Guard Zone Distance (μm)	Mean Measured Section Thickness (μm)	Counting Frame Area (XY) (μm²)	Sampling Grid Area (XY) (μm²)	Number of Sampling Sites	Total Markers Counted	Estimated Population using Mean Section Thickness	Measured Volume (mm³)	Coefficient of Error (Gundersen). m=1	Density (cell/mm³)
17	4	50	13	9	2	13	2,500	122,500	302	114	104,857.07	23	0.10	4,601.98
18	4	50	13	9	2	13	2,500	122,500	299	129	118,507.98	22	0.09	5,399.05
19	4	50	13	9	2	13	2,500	90,000	225	149	100,600.09	12	0.09	8,368.77
20	4	50	13	9	2	13	2,500	90,000	274	130	87,768.15	15	0.09	5,796.95
22	3	50	13	9	2	13	2,500	62,500	444	169	79,301.13	17	0.08	4,583.80
23	4	50	13	9	2	13	2,500	160,000	185	101	121,478.38	18	0.10	6,833.50
26	4	50	13	9	2	13	2,500	62,500	419	111	51,737.45	16	0.10	3,178.09
27	4	50	13	9	2	13	2,500	202,500	250	136	206,708.25	32	0.08	6,518.79
28	4	50	13	9	2	13	2,500	160,000	209	117	140,490.86	21	0.10	6,786.21
29	4	50	13	9	2	13	2,500	40,000	666	136	40,836.98	17	0.09	2,471.84
31	4	50	13	9	2	13	2,500	40,000	779	112	33,628.50	19	0.10	1,732.63
32	4	50	13	9	2	13	2,500	10,000	3,313	151	11,309.97	20	0.08	556.65
34	3	50	13	9	2	13	2,500	62,500	211	135	63,179.29	08	0.09	8,049.10
35	4	50	13	9	2	13	2,500	160,000	283	114	136,173.89	28	0.10	4,841.05
36	4	50	13	9	2	13	2,500	90,000	491	208	140,454.06	28	0.08	5,084.86

Supplementary Table 5. Volume data.

Supplementary Table 5a. Estimated volume of the basolateral nuclear group.

Case	Number of Sections	Section Cut Thickness (μm)	Section Evaluation Interval	Grid Size (μm)	Count	Estimated Area (mm²)	Volume Corrected for Over Projection (mm³)	Coefficient of Error (Gundersen), m=1
17	4	50	13	500	1.953	488.25	310.23	0.02
18	4	50	13	500	1.681	420.25	266.30	0.03
19	4	50	13	500	1.968	492.00	312.58	0.03
20	4	50	13	500	1.754	438.50	278.26	0.03
21	4	50	13	500	2.049	512.25	326.33	0.02
22	4	50	13	500	1.594	398.50	253.76	0.02
23	4	50	13	500	1.344	336.00	212.70	0.04
24	4	50	13	500	1.100	275.00	174.31	0.02
25	4	50	13	500	1.233	308.25	195.71	0.02
26	4	50	13	500	1.505	376.25	239.36	0.02
27	4	50	13	500	1.796	449.00	284.81	0.02
28	4	50	13	500	1.486	371.50	235.99	0.02
29	4	50	13	500	1.775	443.75	282.29	0.02
30	4	50	13	500	1.525	381.25	242.38	0.02
31	4	50	13	500	2.310	577.50	367.69	0.02
32	4	50	13	500	1.483	370.75	235.20	0.03
33	4	50	13	500	1.692	423.00	268.94	0.02
34	4	50	13	500	742	185.50	117.39	0.03
35	4	50	13	500	1.143	285.75	181.16	0.03
36	4	50	13	500	1.328	332.00	210.81	0.02

Supplementary Table 5b. Estimated volume of the cortical nuclear group.

Case	Number of Sections	Section Cut Thickness (µm)	Section Evaluation Interval	Grid Size (µm)	Count	Estimated Area (mm²)	Volume Corrected for Over Projection (mm³)	Coefficient of Error (Gundersen), m=1
17	4	50	13	500	265	66.25	42.15	0.02
18	4	50	13	500	295	73.75	46.85	0.03
19	4	50	13	500	217	54.25	34.46	0.02
20	4	50	13	500	264	66.00	41.98	0.03
21	4	50	13	500	153	38.25	24.24	0.03
22	4	50	13	500	273	68.25	43.31	0.02
23	4	50	13	500	285	71.25	45.31	0.03
24	4	50	13	500	205	51.25	32.50	0.03
25	4	50	13	500	195	48.75	31.04	0.03
26	3	50	13	500	101	25.25	15.79	0.05
27	4	50	13	500	229	57.25	36.43	0.03
28	4	50	13	500	144	36.00	22.89	0.03
29	4	50	13	500	238	59.50	37.84	0.03
30	4	50	13	500	165	41.25	26.16	0.03
31	4	50	13	500	257	64.25	40.78	0.03
32	4	50	13	500	324	81.00	51.31	0.03
33	4	50	13	500	190	47.50	30.06	0.03
34	4	50	13	500	197	49.25	31.26	0.03
35	4	50	13	500	195	48.75	30.89	0.03
36	4	50	13	500	166	41.50	26.40	0.03

Supplementary Table 5c. Estimated volume of the central nuclear group.

Case	Number of Sections	Section Cut Thickness (µm)	Section Evaluation Interval	Grid Size (µm)	Count	Estimated Area (mm²)	Volume Corrected for Over Projection (mm³)	Coefficient of Error (Gundersen). m=1
17	4	50	13	500	140	35.00	22.24	0.03
18	4	50	13	500	153	38.25	24.24	0.02
19	3	50	13	500	74	18.50	11.63	0.05
20	4	50	13	500	137	34.25	21.71	0.03
22	4	50	13	500	103	25.75	16.33	0.04
23	3	50	13	500	83	20.75	13.10	0.04
26	4	50	13	500	104	26.00	16.48	0.04
27	4	50	13	500	223	55.75	35.43	0.03
28	4	50	13	500	211	52.75	33.33	0.03
29	4	50	13	500	130	32.50	20.59	0.03
31	3	50	13	500	104	26.00	16.40	0.03
32	4	50	13	500	198	49.50	31.45	0.03
33	3	50	13	500	71	17.75	11.16	0.05
34	3	50	13	500	37	9.25	5.83	0.06
35	4	50	13	500	174	43.50	27.65	0.03
36	4	50	13	500	243	60.75	38.60	0.03

Supplementary Table 5d. Estimated volume of the amygdala.

Case	Number of Sections	Section Cut Thickness (μm)	Section Evaluation Interval	Grid Size (μm)	Count	Estimated Area (mm^2)	Volume Corrected for Over Projection (mm^3)	Coefficient of Error (Gundersen), m=1
17	4	50	13	500	2,381	595.25	378.36	0.02
18	4	50	13	500	2,149	537.25	340.99	0.03
19	4	50	13	500	2,282	570.50	362.84	0.02
20	4	50	13	500	2,171	542.75	344.58	0.03
21	4	50	13	500	2,223	555.75	354.04	0.02
22	4	50	13	500	1,994	498.50	317.48	0.02
23	4	50	13	500	1,730	432.50	274.16	0.03
24	4	50	13	500	1,320	330.00	209.39	0.02
25	4	50	13	500	1,444	361.00	229.41	0.02
26	4	50	13	500	1,716	429.00	272.96	0.02
27	4	50	13	500	2,157	539.25	342.89	0.02
28	4	50	13	500	1,868	467.00	297.06	0.02
29	4	50	13	500	2,173	543.25	345.91	0.02
30	4	50	13	500	1,707	426.75	271.39	0.02
31	4	50	13	500	2,694	673.50	428.98	0.02
32	4	50	13	500	2,038	509.50	323.66	0.03
33	4	50	13	500	1,987	496.75	315.93	0.02
34	4	50	13	500	1,005	251.25	159.33	0.02
35	4	50	13	500	1,541	385.25	244.60	0.03
36	4	50	13	500	1,758	439.50	279.29	0.02
